# Concurrent consumption of ethanol and corticosterone during adolescence alters neuroimmune sensitivity in Sprague Dawley rats

**DOI:** 10.1016/j.addicn.2025.100218

**Published:** 2025-07-05

**Authors:** Ashley Lutzke, Ariana L. Velazquez, Sarah Trapp, Andrew S. Vore, Hannah E. Burzynski, Maeve E. Johnston, Terrence Deak

**Affiliations:** Developmental Exposure Alcohol Research Center, Behavioral Neuroscience Program, Department of Psychology, Binghamton, NY 13902-6000, United States

**Keywords:** Adolescent ethanol exposure, Corticosterone consumption, Neuroimmune, BBB permeability, Ethanol-induced hypothermia, Poly I:C fever

## Abstract

Chronic stress and alcohol consumption influence various features of neuroimmune reactivity, including neurobehavioral outcomes, induction of neuroimmune genes, blood-brain barrier (BBB) permeability, and core body temperature regulation. The goal of the present studies was to characterize a novel model of chronic ethanol intake in which exogenous corticosterone (CORT), a principal end-product of the Hypothalamic-Pituitary-Adrenal (HPA) axis, was co-consumed in 10 % ethanol. In adolescence (P28–32), pair-housed Sprague-Dawley rats were given a single bottle containing 10 % ethanol with varying concentrations of CORT (0, 25, 50, or 100μg/mL) for 48 h, followed by 48 h of tap water. This four-day sequence was repeated for 12 cycles, ending in early adulthood (P76–80). In Experiment 1, following CORT and ethanol exposure, rats were challenged with restraint stress (30 min), and changes in neuroimmune gene expression were evaluated. Rats with a history of 10 % ethanol + 100μg/mL CORT showed increased interleukin (IL)-6 mRNA expression in the hippocampus relative to water comparators. Experiment 2 probed BBB permeability after perfusion with FITC-labeled dextran (20 kDa), and no changes were found. Remaining experiments evaluated the effects of ethanol/CORT drinking on ethanol-induced hypothermia (Experiment 3) and polyinosinic:polycytidylic acid (Poly I:C)-induced fever (Experiment 4). In females, ethanol consumption (regardless of CORT) delayed return to baseline following the hypothermic response, and in males, 25μg/mL CORT exclusively suppressed fever following Poly I:C challenge. Together, these findings validate a concurrent exposure model of intermittent CORT and ethanol which is translationally relevant to the adolescent experience, and uncovered ethanol- and CORT-induced changes in adult neuroimmune reactivity.

## 1. Introduction

In adults, stressful life experiences and increased levels of alcohol intoxication often coincide. Recent findings indicated that exposure to stressful scenarios in a laboratory setting increased alcohol consumption [1], and high subjective stress during the day increased alcohol consumption that same night [2]. Adolescence is a developmental period characterized by changes in emotional regulation, increased risk-taking, and a heightened motivation to engage in sensation-seeking behaviors [3]. Each of these facets can be stress-inducing, and frequently co-occur with alcohol use [4]. Chang et al. [5] found that stress and alcohol use often coexist in adolescence, and the number of stressful experiences during childhood and adolescence was positively associated with lifetime alcohol consumption. Early initiators of alcohol consumption also reported a higher number of stressful life events, and these individuals demonstrated increased risk for developing alcohol use disorder before reaching 19 years of age [6]. Thus, adolescents are uniquely predisposed to experiencing stress and binge alcohol consumption interactions [7].

Stressful experiences activate a variety of neural and hormonal events, including the release of glucocorticoids (cortisol in humans, corticosterone in rats/mice; CORT) as the end-product of Hypothalamic-Pituitary-Adrenal (HPA) axis activation. For this reason, a growing number of preclinical (rodent) studies have used chronic exposure to CORT in drinking water as a non-invasive approach to emulate the effects of stress across a protracted period of time. Studies using this procedure consistently resulted in anhedonia and helplessness-like behavior [8,9]; decreased motivation for reward when higher effort was required [10]; and decreased levels of evoked CORT following exposure to forced swim and decreased neurogenesis in the dentate gyrus indicative of HPA axis dysfunction [11]. Torregrossa et al. [12] found that chronic CORT exposure during adolescence resulted in increased impulsive choice in a delay discounting task. Beyond effects of CORT exposure on cognitive function, CORT has been directly implicated in voluntary consumption of alcohol. Long-term exposure to CORT in the drinking water increased ethanol consumption in adrenalectomized rats [13], increased ethanol self-administration in male rats [14], and augmented cue-induced reinstatement of ethanol-seeking in female rats [15]. Thus, administration of CORT in the drinking water has emerged as a tractable model that mimics many effects as seen in chronic stress, which may also interact significantly with ethanol reinforcement processes.

Separately, chronic stress and alcohol exposure are potent activators of neuroimmune function, inducing changes in neuroimmune gene expression [16,17], microglial activation [18,19], and blood-brain barrier (BBB) permeability [20,21]. For instance, adolescent stress yielded exaggerated expression of NFκB subunits following an adult lipopolysaccharide (LPS) challenge in rats [17], effects consistent with a sensitized microglial response. Microglia express both mineralocorticoid and glucocorticoid receptors (MR and GR, respectively), providing a direct mechanism by which CORT might influence gene expression in microglia [22]. In fact, when GR was depleted in microglia of mice using a knockout mouse line, chronic stress no longer induced a hyper-activated state compared to their WT counterparts [23]. Similar to stress-induced sensitization of microglia, Walter et al. [24] found that chronic ethanol exposure sensitized the expression of CD11b, a commonly used marker of microglial activation, following an acute stress challenge, and Siemsen et al. [25] concluded that adult LPS challenge induced a distinct, exaggerated activation state in male rats with chronic ethanol history compared to rats without an ethanol history. In addition, Gano et al. [26] found that adolescent intermittent ethanol (AIE) exposure sensitized fever in male rats following a polyinosinic:polycytidylic acid (Poly I:C) challenge in adulthood. Thus, glucocorticoid signaling is a shared contributor to both stress and ethanol-mediated changes in microglial expression of neuroimmune genes. However, the potential long-lasting impact of concurrent ethanol consumption and chronic CORT administration have not been evaluated systematically.

The BBB is another component of the neuroimmune system that is negatively impacted by both stress and ethanol exposure. The BBB serves as a gating mechanism, protecting the CNS from harmful molecules circulating in the periphery and consists of microglia, astrocytic end-feet, pericytes, and endothelial cells that line the neurovasculature [27]. Interestingly, decreased tight junction protein expression, tight junctions acting as the primary physical barriers between the periphery and CNS, and consequent increased BBB permeability have now been reported after several chronic stress procedures [20,28,29]. In a similar manner, ethanol exposure decreased tight junction protein expression and increased BBB permeability, effects that were associated with increased expression of inflammatory factors [30]. Rubio-Araiz et al. [21] showed that repeated ethanol exposure increased BBB permeability in WT mice but not TLR4 knockout mice, supporting these effects may require TLR4-mediated signaling. Lastly, AIE increased BBB permeability in male but not female rats [31]. In sum, both stress and ethanol independently increase BBB permeability, but the effects of concurrent ethanol and CORT consumption on BBB permeability is unknown.

With this in mind, the overarching goal of the present studies was to characterize a novel model of chronic ethanol intake in which CORT was co-consumed with a (10 %) ethanol solution throughout the adolescent period to evaluate the potential for additive or synergistic effects. Though a number of studies have demonstrated effects of chronic stress or alcohol on cognition and neuroimmune function, very few have characterized the potential summative changes co-exposure of ethanol and CORT may produce. Our analysis included an evaluation of (a) concentration-dependent effects of CORT on ethanol intake in females (Exp 1); (b) assessment of restraint-evoked changes in neuroimmune gene expression and CORT reactivity after a period of protracted abstinence; (c) assessment of BBB permeability after concurrent ethanol and CORT intake throughout adolescence in both sexes (Exp 2); (d) analysis of ethanol-induced hypothermia and evoked changes in gene expression (Exp 3); and (e) evaluation of fever following Poly I:C challenge (Exp 4).

## 2. Methods

### 2.1. Subjects

Male and Female Sprague-Dawley rats were bred in-house from breeders originally acquired from Envigo. Litters were culled to 10 pups (4–6 of each sex) within 24 h of birth; this was recorded as postnatal day 1 (P1). At P21, rats were weaned and housed with same-sex conspecifics from a different litter. No >1–2 pups from each litter were assigned to each experimental group to control for litter effects [32]. The colony room in which rats were kept was maintained at 22 ± 1°C on a 12:12 light:dark cycle with lights turning on at 0700. Throughout the experiments, rats were pair-housed in standard plexiglass cages including chew blocks and nest packs for enrichment and had ad libitum access to food and water. Per experimental guidelines, rats did not have ad libitum access to water during “on” days of the drinking paradigm. Prior to experimentation, rats were handled on two consecutive days for 2–3 min to acclimate them to experimenters. Rats were treated in accordance with Public Health Services (PHS) policy and all experimental protocols were approved by Binghamton University’s Institutional Animal Care and Use Committee (IACUC).

### 2.2. Single bottle intermittent ethanol and corticosterone (CORT) drinking procedure

**Fig. 1.**
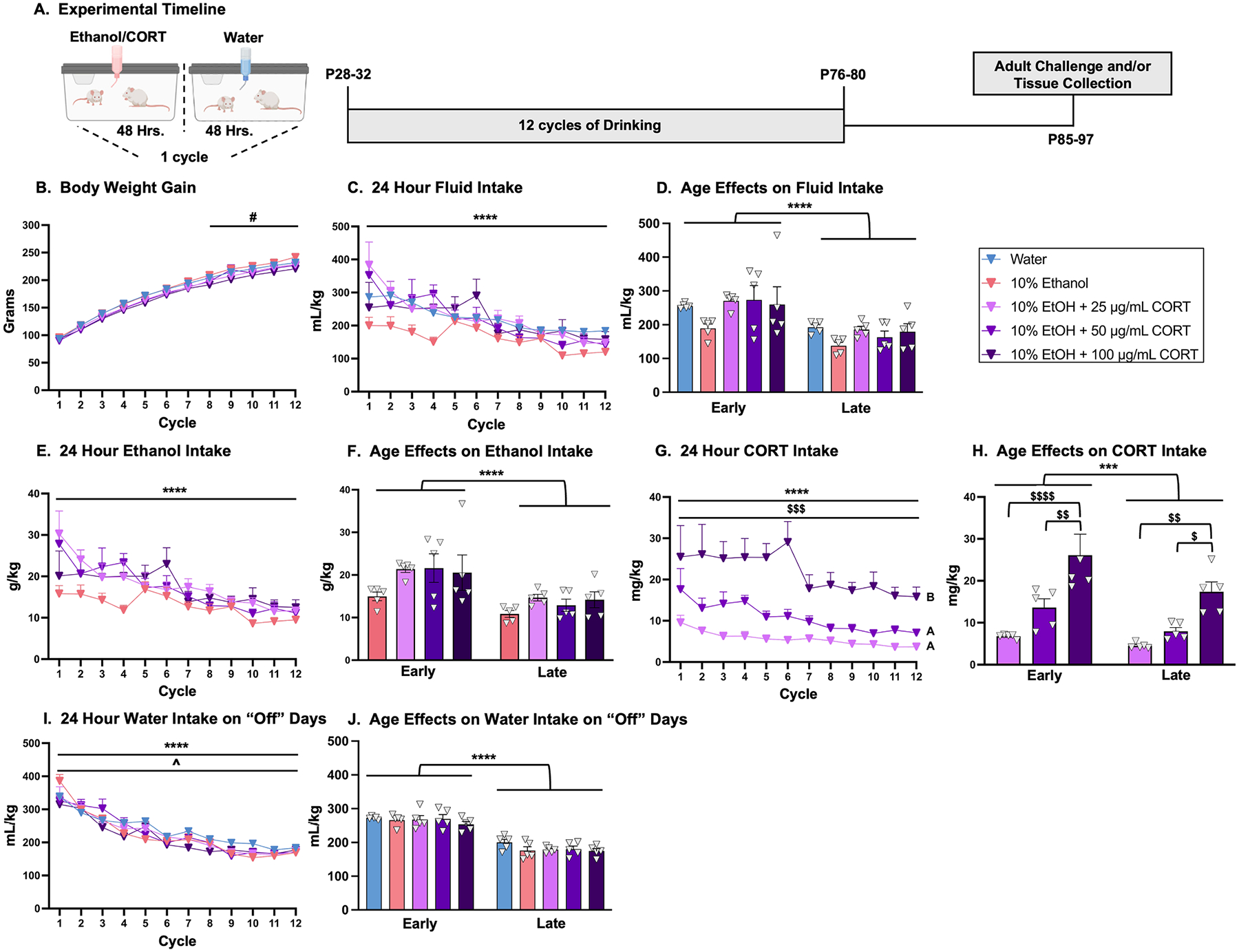
Rats consumed meaningful concentrations of 10 % ethanol and CORT. (A) Experimental timeline. Rats had access to 10 % ethanol +/− CORT or water depending on group for 48 hrs followed by 48 hrs of access to water. This 2-on 2-off cycle was repeated 12 times spanning adolescence and early adulthood. 10–12 days later, rats underwent an adult challenge (Exp’s 1, 3, 4) and/or tissue collection. (B) Rats that consumed ethanol + 100 μg/mL CORT experienced slower weight gain than all other groups during cycles 8–12. (C) Fluid consumption (mL/kg) decreased throughout the drinking paradigm for all groups. (D) To evaluate differences in consumption during early versus late cycles of consumption, data were binned by taking the average mL/kg intake of cycles 1–6 (early) and cycles 7–12 (late). Rats consumed more fluid during early versus late cycles of consumption. (E) Ethanol consumption (g/kg) decreased throughout the drinking paradigm for all ethanol-consuming groups. (F) Data were again binned into early versus late cycles of consumption, and this supported increased ethanol intake in early versus late cycles of consumption. (G) CORT intake (mg/kg) was calculated throughout the drinking paradigm, and CORT consumption decreased throughout the drinking paradigm. Rats that consumed ethanol + 100 μg/mL CORT also consumed significantly more CORT than both other CORT-consuming groups. (H) Data were binned into early versus late cycles of consumption, and rats consumed more CORT during early compared to late cycles of consumption. The rats that consumed ethanol + 100 μg/mL CORT also consumed more CORT than both other groups regardless of cycles of consumption. (I) Fluid intake was also recorded for the 48 hr water-access period of each cycle. Water intake during the “off” days decreased as the drinking paradigm progressed, and water consumption patterns differed for the groups throughout the drinking paradigm. Data were binned into early versus late cycles of consumption (J) to further understand the significant interaction. Water intake during the “off” days was higher in early versus late cycles of consumption. # indicates 100 μg/mL CORT different from all other groups, *p* < 0.05; **** indicates main effect of cycle or cycles of consumption, *p* < 0.0001; $$$$ indicates main effect of CORT, *p* < 0.0001; $$$ indicates main effect of CORT, *p* < 0.001; $$ indicates main effect of CORT, *p* < 0.01; $ indicates main effect of CORT, *p* < 0.05; and ^ indicates cycle × ethanol interaction, *p* < 0.05.

Beginning at P28–32, rats were exposed to an intermittent ethanol consumption procedure that was recently shown to sensitize neuroimmune gene expression and stress sensitivity [33] and influence microglial function [34]. This procedure routinely yields Blood Ethanol Concentrations (BECs) ranging from 40–120 mg/dl within the first 3 hr of bottle exposure, suggesting that most rats surpassed criteria for binge drinking (80 mg/dL; [35]). As shown in Fig. 1A, rats were given a single bottle containing water or 10 % ethanol combined with CORT (0, 25, 50 or 100 μg/mL CORT) as the sole source of fluids for 48 hrs followed by access to tap water for the next 48 hrs. Each 2-day on, 2-day off period constituted 1 cycle, and all rats underwent 12 cycles spanning the ages of P28-P32 (early adolescent initiation) to P76–80 (young adult termination). These concentrations of CORT were selected because the chronic CORT in the drinking water model has been characterized when using 50 μg/mL CORT, which resulted in increased anhedonia [8,9]. The current experiments selected concentrations that were both greater (100 μg/mL CORT) and less (25 μg/mL CORT) than this concentration. Bottles were weighed prior to initial placement on the cages as well as 24 and 48 hrs later (approximately 1500 hr ± 1 hour) to estimate daily intake during the “on” days. One “drip bottle” for each solution was also present on each rack of rats and was weighed in the same manner as the experimental bottles to account for fluid loss due to removal and replacement of bottles on cages. Drip values were subtracted from the daily intake volume for each cage. Daily (24 hr.) intake was recorded per cage (2 rats) rather than per individual rat to avoid social isolation, a stimulus known to have a profound impact on HPA axis function. To account for this, daily intake was divided by the sum of the body weight for both rats in the cage to estimate daily intake on a g/kg basis as in our prior work [33].

### 2.3. Drinking solution preparation

Depending on experiment and group, rats were given water, 10 % ethanol, and CORT (25, 50, or 100 μg/mL) in standard drinking bottles. All solutions were made fresh on the first day of each cycle. Water bottles were filled using tap water, and the 10 % ethanol solution was made by diluting 95 % ethanol stock solution (VWR, 71002–510) to 10 % ethanol in tap water. CORT (Sigma, 27840) was first dissolved in 95 % ethanol due to its hydrophobic nature and then diluted using tap water to achieve the targeted concentration of CORT and ethanol.

### 2.4. Brain processing for assessment of neuroimmune gene expression

Brain regions of interest were collected by punching frozen sections on a cryostat. ROIs included the paraventricular nucleus of the hypothalamus (PVN) and hippocampus (HPC) since both structures are highly responsive to stress and alcohol [17,36,37]. Paxinos and Watson’s 2nd Edition Brain Atlas was used to guide the isolation of each region. Each region was collected and stored in RNAse-free 2 mL tubes at −80°C until RNA extraction. RNA was extracted by adding 5 mm stainless steel beads and 500 μL of Trizol to the centrifuge tubes and bead pulverized using a TissueLyser II (Qiagen, Valencia, Ca, USA). Homogenized samples were then purified using a RNEasy mini kit (Qiagen, 74106) to extract the RNA and eluted in 65°C RNAse-free water. Nanodrop (ThermoScientific, Waltham, MA, USA) was then utilized to evaluate the RNA quality and concentration, and to normalize samples. cDNA synthesis was performed using a QuantiTect reverse transcription kit (Qiagen, 205314). RT-PCR was then conducted as previously described [33] using primer sets provided in Table 1. Samples were run in triplicate and underwent denaturation, annealing, and extension over a total of 40 cycles in a 384-well plate using a CFX384 real-time PCR detection system (Bio-Rad). Data from the housekeeper, (GAPDH) was analyzed as expression relative to the ultimate control (rats in the water group) to ensure there are no group differences in housekeeper gene expression. Output data for targets of interest were analyzed by normalizing each sample to the expression of the housekeeper (GAPDH) and then relative to the ultimate control (rats in the water group) using the 2^ΔΔC(t)^ method [33].

### 2.5. Measurement of plasma CORT

Total plasma CORT was measured in plasma using a commercially-available EIA kit (Enzo Life Sciences, ADI-901–097) according to manufacturer instruction, with one exception. Samples were heat-inactivated in a 75°C water bath for one hour to denature endogenous corticosteroid binding globulin (CBG), because heat denaturation has proven to be more effective than use of the steroid displacement reagent that is provided with the kit (see Spencer & Deak [38] for discussion).

### 2.6. Measurement of core body temperature

To record core body temperature, remote telemetry microchips (Unified Information Devices; UID) were implanted in each rat. Rats were briefly anesthetized using isoflurane (3 %), and then a microchip trocar (UID) was used to implant the microchip into the left flank of each rat in a minimally invasive manner. Each rat was immediately scanned using a handheld microchip reader (UID) to ensure functionality of the microchip, and individual identifications were recorded. During experiments in which core body temperature was recorded, the day before the adult challenge, cages were spread apart to allow for scanning of individual rats without disturbing the cage i.e., scans were taken using the handheld microchip reader from outside the homecage within the colony rooms to minimize the influence of the experimenter on rat core body temperature.

### 2.7. Statistical analysis

All data were visualized and analyzed using GraphPad Prism (GraphPad Software Inc., California) or Statistica (Statsoft). In all experiments, for repeated measures analyses and significant 2- or 3-way interactions, Statistica (TIBCO Data Science; Palo Alto, California, United States) was used to further clarify group differences. Consumption data throughout the drinking procedure (mL/kg and g/kg across cycle) were analyzed using a mixed ANOVA including time as a repeated measures variable. Similarly, a mixed ANOVA was also used to evaluate consumption data (mL/kg, g/kg, and mg/kg) binned into early versus late cycles of consumption. To avoid imputing data or excluding entire animals from the analysis when an individual data point was missing (i. e. a bottle leaked one day), mixed-effects model analysis was used when necessary and is specified in the results. Additionally, when analyzing only the repeated measures components of all designs, sphericity was not assumed and the Geisser and Greenhouse method was used to correct for lack of sphericity. To evaluate differences in gene expression (RT-PCR), organ weights, and plasma CORT in Experiment 1, a one-way ANOVA was used, and *post-hoc* analyses were performed using Tukey’s HSD to evaluate group differences. To analyze BBB permeability data in Experiment 2, a two-way ANOVA was used with Tukey’s HSD as a *post-hoc* analysis. In Experiment 3, a 2 × 2 ANOVA was used to evaluate differences in BECs and gene expression (RT-PCR). To evaluate differences in ethanol-induced hypothermia (raw body temperatures and change from baseline), a mixed ANOVA was used to evaluate differences in baseline temperatures and core body temperatures following the ethanol challenge separately. Similarly, in Experiment 4, a mixed ANOVA was used to separately evaluate baseline differences in core body temperature; the return to baseline post-injection; the overall fever response; the rise in fever; and the fall in fever. Peak fever and peak change were analyzed by evaluating the peak raw temperature or peak change from baseline for each individual rat. This was analyzed using a 2 × 2 ANOVA. Alpha level was set at 0.05 for all statistical tests. Grubbs’ test was used to evaluate and remove outliers. In Experiment 1, three rats were also removed from RT-PCR statistical analyses of the PVN (two from the water group and one from the 10 % ethanol + 100 μg/mL CORT group) due to insufficient housekeeper (GAPDH) data. In addition, in Experiment 4, there were four rats that were deemed as “nonresponders” to Poly I:C: two in the (water, 25 μg/mL CORT) group; one in the (10 % ethanol, none) group; and one in the (10 % ethanol, 25 μg/mL CORT) group that were consequently excluded from all analyses. This was determined because the stress-induced spike in temperature post-injection exceeded the fever response induced by Poly I:C for each of these rats.

### 2.8. Experiment 1 – adolescent ethanol and CORT effects on adult stress reactivity

In humans, stressful circumstances are often simultaneously or immediately followed by increased alcohol consumption [1]. Importantly, this is true for both AUD patients and nonclinical social drinkers [2]. In rodent studies, however, stress exposure yields variable effects with some studies showing increased [39–42], decreased [43], or no effect [44] on ethanol consumption. The goal of Experiment 1 was to (a) evaluate whether CORT would increase consumption of ethanol; (b) characterize the impact of combined ethanol and CORT administration on body and organ weights; and (c) determine whether chronic ethanol and CORT would influence restraint-induced expression of neuroimmune genes and HPA reactivity. The design of this experiment included five groups (*N* = 50, *n* = 10), water or 10 % ethanol with escalating concentrations of CORT (0, 25, 50, and 100 μg/mL). Adolescent, female Sprague-Dawley rats underwent the single bottle intermittent ethanol and CORT drinking procedure. It was hypothesized *a priori* that the lowest concentration of CORT (25 μg/mL) would influence 10 % ethanol consumption while higher concentrations would not, so a separate analysis was conducted including only the 25 μg/mL CORT group. Rats then remained undisturbed for 10–11 days in which they had ad libitum access to food and water. This period of time was included to ensure any residual neuroimmune effects of acute ethanol withdrawal had fully abated. Acute restraint was then performed in a Plexiglas restraint tube for 30 min and rats were then euthanized by rapid decapitation. Trunk blood was collected into EDTA coated glass tubes for later assessment of plasma CORT. Thymus, spleen, and adrenal glands were collected and flash-frozen on dry ice before being weighed, whereas brains were immediately flash frozen in 2-methylbutane and stored at − 80°C until later analysis.

### 2.9. Experiment 2 – adolescent ethanol and CORT effects on BBB permeability

Sex differences in HPA axis function and reactivity are well-established, with females generally exhibiting higher basal and stress-evoked CORT responses than males [38,45,46]. In addition, recent work from our lab showed that adolescent intermittent ethanol (AIE) exposure using a typical ethanol gavage procedure led to male-specific changes in BBB permeability later in adulthood [31]. Thus, the goals of Experiment 2 were to evaluate whether coadministration of ethanol and CORT in the drinking water during adolescence would (a) increase ethanol consumption in both sexes, and (b) produce sex-specific changes in BBB reactivity. This experiment utilized a 2 sex (male, female) × 2 drinking condition (water, 10 % ethanol + 25 μg/mL CORT) factorial design (*N* = 40, *n* = 10). The drinking procedure was performed at the same ages for Experiment 1, followed by a recovery period of 21 days. Note that the post-ethanol recovery period was extended from 10–11 days (Experiment 1) to 20–21 days (Experiment 2) to match our prior work in which BBB permeability was assessed after ethanol gavage [31].

#### Perfusions with 20 kDa FITC-dextran.

For dextran perfusions, a 10 mg/mL dextran solution was prepared using 20 kDa FITC-dextran diluted into cold 0.1 M phosphate buffered saline (PBS). Because 20 kDa FITC-dextran is light-sensitive, all steps of the dextran perfusions took place in the dark under red light only. Rats were first euthanized with Fatal-Plus solution (Vortech Pharmaceutical; Pentobarbital Sodium at 390 mg/mL concentration) at a dose of 1 mL/kg i.p. Using a peristaltic pump, 16 mL of dextran solution was perfused at a rate of 12 mL/min. Once perfusions were complete, brains were collected and kept in paraformaldehyde (PFA) for 72 h to post-fix the tissue, then cryoprotected with 10 % glycerol and 20 % glycerol consecutively for 24 hrs each. All brains were then frozen in 2-methylbutane at −20°C for 2 min and gently wrapped in foil and stored at −20°C.

#### Image analysis for BBB permeability.

Image analysis procedures were adapted from Natarajan et al. [47] as described in our recent work [31]. Brains were sliced on a cryostat at 100 μm and immediately cover slipped using Prolong Diamond Antifade Mountant with DAPI (Thermofisher Scientific, P36970). Slides were allowed to set for 24 hrs in the dark and stored at 4°C to minimize exposure to light. Slides were then imaged using a Leica TCS SP8 Confocal microscope at 10x using LASX navigation software. Regions of interest (nucleus accumbens, cingulate prefrontal cortex, amygdala, and hippocampus) were selected and outlined for imaging, and individual tiles were 512×512 resolution at a bidirectional scanning speed of 400 hz. A Z-stack of approximately 100 μm was captured, and LASX software was used to generate max projections which compiled all signal captured in the 100-micron Z-stack into a 2-dimensional image. ImageJ software was then used to remove fluorescence from the blood vasculature so that only ambient fluorescence reflecting FITC-dextran that had leaked from the vasculature into the surrounding brain tissue was analyzed. Lastly, regions of interest were generated individually for each image and relative fluorescence units (RFU) was determined by ImageJ. For the amygdala and hippocampus, the same images were used to determine RFU of their respective subregions (basolateral, medial, and central amygdala; CA1, CA2, CA3, and dentate gyrus of the hippocampus).

### 2.10. Experiment 3 – adolescent ethanol and CORT effects on ethanol-induced hypothermia

Exposure to high doses of ethanol consistently results in a hypothermic response [48]. Interestingly, Carreño et al. [49] found that ethanol-induced hypothermia was blocked by pretreatment of dexamethasone, a GR agonist, supporting that HPA axis functionality, specifically GR translocation, may modulate the hypothermic response to high doses of ethanol. In addition, Taylor et al. [50] found that in ethanol-dependent rats, females displayed a greater hypothermic response to ethanol compared to males. Therefore, the goals of Experiment 3 were to determine whether history of ethanol +/− CORT consumption would alter (a) the hypothermic response to an ethanol challenge, and (b) mRNA cytokine expression in female Sprague-Dawley rats. Experiment 3 utilized a 2 Ethanol (water, 10 % ethanol) × 2 CORT (none, 25 μg/mL CORT) factorial design (*N* = 48, *n* = 12). The adolescent drinking paradigm was followed as explained above (Section 2.3), and rats remained undisturbed for one week to ensure they were not in withdrawal. Microchips to measure core body temperature were then implanted (Section 2.5), and rats were allowed five to six days of post-operative recovery. At P88–93, baseline temperature scans were taken at 0815 hr, 0900 hr, and 0945 hr. All rats were then administered an ethanol challenge (2.0 g/kg, i.p.) at 1000 hr +/− 30 min, and temperatures were individually recorded 30-, 60-, 90-, and 120-min post-injection to measure the hypothermic response to ethanol. Immediately following the final temperature scan, rats were rapidly decapitated to collect trunk blood and brains. Trunk blood was collected in vacutainers and centrifuged for 10 min to allow for separation of plasma and whole blood cells. Plasma was then collected and used to evaluate differences in blood ethanol concentrations (BECs) and plasma CORT. Whole brains were punched and RT-PCR was used to assess for changes in neuroimmune gene expression in regions of interest.

#### Blood ethanol concentrations (BECs).

The Analox AM1 instrument was used to measure BECs for each sample. Briefly, plasma samples were thawed at room temperature, vortexed, and underwent a brief spin (30 s) to remove any sample from the cap of the 1.5 mL tubes. The instrument was set up according to manual guidelines, and the instrument was calibrated every ten samples.

### 2.11. Experiment 4 – adolescent ethanol and CORT effects on Poly I:C-induced fever

Both ethanol exposure and chronic stress have been shown to sensitize the neuroimmune response following challenge [17,24,25]. In our lab, we found that AIE (adolescent intermittent ethanol exposure via gavage) sensitized the fever response to Poly I:C in male but not female Sprague-Dawley rats [26]. The goals of Experiment 4 were to determine whether (a) the sensitized fever response following Poly I:C challenge [26] would translate to a moderate ethanol consumption model, and (b) concurrent consumption of ethanol and CORT would impact fever. This experiment utilized a 2 Ethanol (water, 10 % ethanol) × 2 CORT (none, 25 μg/mL CORT) factorial design (*N* = 43, *n* = 8–10). Male, Sprague Dawley rats underwent the adolescent drinking paradigm (Section 2.2), and then remained undisturbed for four days. Microchips were then implanted (Section 2.5), and rats were allowed two days of recovery. During the two-day recovery period and for the remaining duration of the experiment, rats were single housed to prevent huddling behavior from obscuring the magnitude of the evoked fever. At P85–90, baseline temperature scans were taken at 0815 hr, 0900 hr, and 0945 hr. All rats were then administered Poly I:C (4.0 mg/kg, i.p.) at 0945 hr immediately following the last baseline temperature scan. Temperatures were then individually recorded every 30 min for eight hours total to determine differences in fever response.

## 3. Results

### 3.1. Experiment 1: adolescent ethanol and CORT effects on adult stress reactivity

#### Body Weight.

During the single bottle intermittent ethanol and CORT drinking procedure, rat weights were recorded at the start of each 4-day cycle. A mixed 5 × 12 ANOVA with one between subjects variable (group) and one repeated measures variable (cycle) revealed a main effect of cycle, *F*_(2.6, 116.4)_ = 2139, *p* < 0.0001, demonstrating that all groups gained weight throughout the drinking procedure. Analysis also revealed a significant cycle × group interaction, *F*_(44, 495)_ = 1.524, *p* = 0.0193, with *post-hoc* analyses revealing that from cycles 8–12, rats consuming 10 % ethanol + 100 μg/mL CORT showed moderately suppressed body weight gain relative to water comparators (Fig. 1B).

#### Total Fluid Intake (mL/kg).

A mixed 5 × 12 ANOVA with one between-subjects variable (group) and one repeated measures variable (cycle) of fluid intake (mL/kg) during “on days” (i.e., days in which the experimental solution was available) revealed a main effect of cycle, F_(3.7, 73.4)_ = 17.55, *p* < 0.0001, demonstrating that consumption decreased throughout the drinking paradigm independent of experimental condition (Fig. 1C). Data were also binned into early (cycles 1–6) vs. late (cycles 7–12) cycles of consumption to evaluate age-specific effects. A mixed 5 × 2 ANOVA with one between-subjects variable (group) and one repeated measures variable (cycles of consumption) was conducted. This analysis revealed a main effect of cycles of consumption, *F*_(1, 20)_ = 56.93, *p* < 0.0001; rats had higher consumption during early versus late cycles of consumption regardless of group (Fig. 1D). No significant main effect of group or cycles of consumption × group interaction was observed.

#### 24-hr Ethanol Intake (g/kg).

To evaluate the effects of concurrent CORT and ethanol consumption, ethanol intake as a function of body weight (g/kg) was analyzed using a mixed 5 × 12 ANOVA with one between-subjects variable (group) and one repeated measures variable (cycle). There was a significant main effect of cycle, *F*_(3.6, 58)_ = 12.61, *p* < 0.0001, supporting that consumption differed throughout the drinking procedure independent of group (Fig. 1E), with *post-hoc* analyses revealing a decline in ethanol intake as a factor of cycle. It was hypothesized *a priori* that the lowest concentration of CORT (25 μg/mL) would influence ethanol intake, so a mixed 2 × 12 ANOVA with one between-subjects variable (group) and one repeated measures variable (cycle) was performed including only the 10 % ethanol and 10 % ethanol + 25 μg/mL CORT. This analysis revealed a significant main effect of cycle, *F*_(3.4, 26.9)_ = 6.291, *p* = 0.0017, supporting that rats again differed in their ethanol intake regardless of group. A significant main effect of group was also revealed, *F*_(1, 8)_ = 23.40, *p* = 0.0013, indicating that the 10 % ethanol + 25 μg/mL CORT group consumed significantly more ethanol (g/kg) than the 10 % ethanol group across the entire timeline (Fig. 1E). Similar to fluid intake (mL/kg), ethanol intake was also binned into early (cycles 1–6) and late (cycles 7–12) cycles of consumption. The mixed 4 × 2 ANOVA with one between-subjects variable (group) and one repeated measures variable (cycles of consumption) revealed a significant main effect of cycles of consumption, *F*_(1, 16)_ = 41.08, *p* < 0.0001, such that rats had higher consumption during early compared to late cycles of consumption regardless of group (Fig. 1F). No significant main effect of group or cycles of consumption × group interaction was noted.

#### 24-hr CORT intake.

Estimates of CORT intake were only calculated for experimental groups in which CORT was included in the drinking water. A mixed 3 × 12 ANOVA with one between-subjects variable (group) and one repeated measures variable (cycle) for CORT intake (mg/kg) revealed a significant main effect of cycle, *F*_(2.5, 30.4)_ = 7.889, *p* = 0.0009, demonstrating reduced CORT intake throughout the study (Fig. 1G). *Post-hoc* analyses showed that CORT intake showed a modest decline across cycles for all groups. As expected, there was also a significant main effect of group (CORT concentration), *F*_(2, 12)_ = 14.04, *p* = 0.0007, which yielded a concentration-dependent increase in CORT intake (100 μg/mL CORT group > 50 μg/mL CORT and 25 μg/mL CORT) regardless of cycle (Fig. 1G). Similar to the earlier data analysis parameters, CORT intake data were binned to analyze differences in CORT consumption during early (cycles 1–6) versus late (cycles 7–12) cycles of consumption. A mixed 3 × 2 ANOVA with one between-subjects variable (group) and one repeated measures (cycles of consumption) revealed a significant main effect of cycles of consumption, *F*_(1, 12)_ = 18.69, *p* = 0.001, such that rats consumed more CORT during early compared to late cycles of consumption regardless of group (Fig. 1H). There was also a significant main effect of group, *F*_(2, 12)_ = 14.04, *p* = 0.0007, such that the 10 % ethanol + 100 μg/mL CORT group consumed more CORT than both the 10 % ethanol + 25 μg/mL CORT and the 10 % ethanol + 50 μg/mL CORT groups regardless of cycles of consumption (Fig. 1H). There was no significant difference in CORT consumption between the 10 % ethanol + 25 μg/mL CORT and 10 % ethanol + 50 μg/mL CORT groups.

#### Water consumption during “off” days.

Water intake (mL/kg) was evaluated during “off days” to determine whether concurrent consumption of ethanol and CORT might produce “rebound” effects on water intake. To test this, a mixed 5 × 12 ANOVA with one between-subjects variable (group) and one repeated measures variable (cycle) revealed a significant main effect of cycle *F*_(4.1, 83)_ = 105.5, *p* < 0.0001, indicating that water consumption differed throughout the procedure, showing a similar decline across cycles/age as was observed during “on” days. There was also a significant cycle × group interaction, *F*_(44, 220)_ = 1.489, *p* = 0.0338, indicating different water consumption patterns between groups across the drinking procedure (Fig. 1I). To understand the significant cycle × group interaction, data were again binned into early versus late cycles of consumption on the “off” days. A mixed 5 × 2 ANOVA with one between-subjects variable (group) and one repeated measures (cycles of consumption) revealed a significant main effect of cycles of consumption, *F*_(1, 20)_ 265.8, *p* < 0.0001, indicating that rats consumed more water during the “off” days in early versus late cycles of consumption (Fig. 1J). There was no significant main effect of group or interactions between cycles of consumption × group.

#### Plasma CORT and Organ Weights Following Restraint Stress.

CORT analyses were performed to assess the effects of intermittent ethanol and CORT exposure during adolescence on later stress sensitivity, assessed by 30 min of restraint. Using a one-way between-subjects ANOVA, no effects on peak CORT release were observed between groups (Table 2). Adrenal glands, spleen, and thymus were also weighed to evaluate the effects of intermittent ethanol and CORT exposure on peripheral organs involved in endocrine and immune function. No significant effects were observed between groups for any of these measures regardless of whether raw tissue weights (mg) or tissue weights adjusted to body weight (mg/kg) were analyzed (Table 2).

#### Gene Expression in the PVN and Hippocampus.

RT-PCR was performed on brain punches of the PVN and HPC to investigate changes in mRNA expression following intermittent ethanol and CORT intake followed by restraint stress. For both the PVN and HPC, a one-way between-subjects ANOVA revealed there were no significant differences in housekeeper gene (GAPDH) expression between groups (Table 3). The ANOVA did not reveal significant changes in gene expression for IL-1β, IL-6, IκBɑ, Iba1, or GFAP in the PVN (Table 3). In the HPC, there was a significant difference in IL-6 gene expression, *F*_(4, 42)_ = 2.793, *p* = 0.0382, such that expression was greater in the 10 % ethanol + 100 μg/mL CORT compared to the water group (Table 3). There were no other significant differences in gene expression (IκBɑ or GFAP) in the HPC (Table 3). Additionally, PCR was used to assess MR and GR expression due to previous research suggesting that chronic stress and alcohol exposure decrease MR and GR expression in the brain [51–53]. The ANOVA revealed no significant differences in expression of MR or GR in the PVN or HPC (Table 3).

### 3.2. Experiment 2: concurrent adolescent ethanol and CORT effects on BBB permeability

#### Body Weight.

**Fig. 2.**
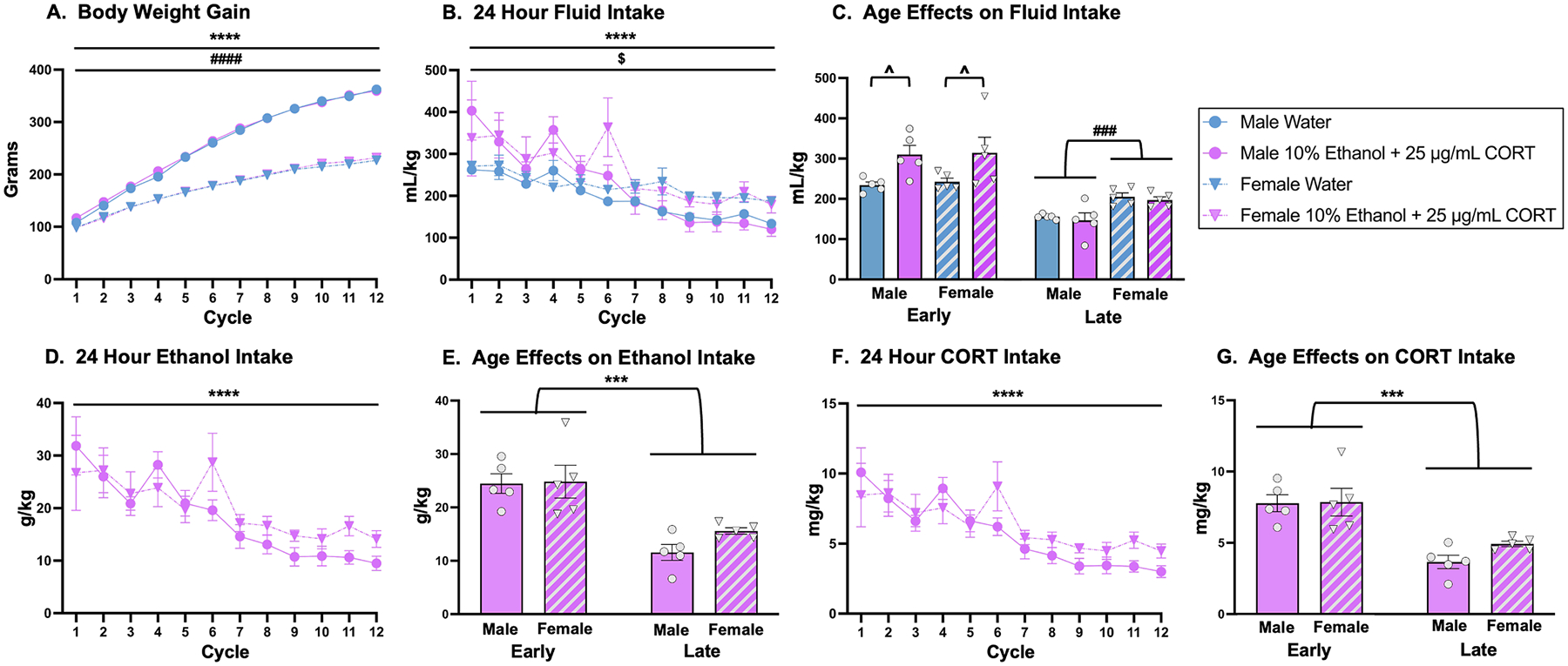
Male and female rats consumed more ethanol + CORT compared to water during early cycles of consumption exclusively. (A) All rats gained weight throughout the drinking paradigm, and male rats weighed more and gained weight more quicky than female rats. (B) Fluid consumption (mL/kg) decreased throughout the drinking paradigm for all groups, and males decreased their consumption more quickly than female rats. Rats in the ethanol + 25 μg/mL CORT consumed more fluid than rats in the water group regardless of sex. (C) To evaluate differences in consumption during early versus late cycles of consumption, data were binned by taking the average mL/kg intake of cycles 1–6 (early) and cycles 7–12 (late). Rats consumed more fluid during early versus late cycles of consumption. During early cycles of drinking exclusively, rats in the ethanol + 25 μg/mL CORT consumed more fluid than rats in the water group regardless of sex. Female rats also consumed more than male rats exclusively during the late cycles of consumption. (D) Ethanol consumption (g/kg) decreased throughout the drinking paradigm for both males and females. (E) Data were again binned into early versus late cycles of consumption, and this supported increased ethanol intake in early versus late cycles of consumption. (F) CORT intake (mg/kg) was calculated throughout the drinking paradigm, and CORT consumption decreased throughout the drinking paradigm. (G) CORT intake data were binned into early versus late cycles of consumption, and rats consumed more CORT during early compared to late cycles of consumption. **** indicates main effect of cycle or cycles of consumption, *p* < 0.0001; *** indicates main effect of cycle or cycles of consumption, *p* < 0.001; #### indicates cycle × sex interaction, *p* < 0.0001; ### indicates cycle × sex interaction, *p* < 0.001; ^ indicates a cycles of consumption × drinking history interaction, *p* < 0.05; and $ indicates main effect of drinking history, *p* < 0.05.

Similar to the first experiment, body weights were recorded at the beginning of each cycle of the drinking procedure. A mixed 2 × 2 × 12 ANOVA with two between-subjects variables (sex and drinking history) and one repeated measures variable (cycle) was used to analyze these data. The analysis revealed a significant main effect of cycle, *F*_(2.5, 88.6)_ = 4132, *p* < 0.0001, supporting that rats gained weight as they aged (Fig. 2A). As expected, there was also a significant main effect of sex, *F*_(1, 36)_ = 654.8, *p* < 0.0001, and a significant cycle × sex interaction, *F*_(11, 396)_ = 448.6, *p* < 0.0001, such that males weighed more and gained weight more quickly than female comparators (Fig. 2A). The three-way interaction (sex × drinking history × time) was also significant, *F*_(11, 396)_ = 2.642, *p* = 0.0028, and *post-hoc* analyses revealed that weights of the male 10 % ethanol + 25 μg/mL CORT group differed from both female groups, but not the direct male comparator (Fig. 2A).

#### Total Fluid Intake (mL/kg).

A mixed 2 × 2 × 12 ANOVA with two between-subjects variables (sex and drinking history) and one repeated measures variable (cycle) was used to evaluate differences in total fluid intake. Analysis revealed a significant main effect of cycle, *F*_(4.1, 65.9)_ = 18.66, *p* < 0.0001, in which rats again consumed less fluid as the cycles progressed (Fig. 2B). There was also a significant main effect of drinking history, *F*_(1, 16)_ = 4.545, *p* = 0.0489, in which rats in the 10 % ethanol + 25 μg/mL CORT drank significantly more than rats that consumed water (Fig. 2B). A significant cycle × sex interaction was also observed, *F*_(11, 176)_ = 1.959, *p* = 0.0351, with *post-hoc* analyses revealing that males and females consumed comparable amounts of fluid in the early cycles but not the late cycles (Fig. 2B). A significant cycle × drinking history interaction was also observed, *F*_(11, 176)_ = 3.286, *p* = 0.0004, and *post-hoc* analyses supported that rats that consumed 10 % ethanol + 25 μg/mL CORT decreased their consumption across cycles earlier compared to rats that consumed water across the cycles (Fig. 2B). When data were binned into early (cycles 1–6) versus late (cycles 7–12) cycles of consumption, a mixed 2 × 2 × 2 ANOVA with two between-subjects variables (sex and drinking history) and one repeated measure (cycles of consumption) revealed a significant main effect of cycles of consumption, *F*_(1, 16)_ = 96.18, *p* < 0.0001, with higher consumption in the early versus late cycles regardless of drinking history (Fig. 2C). A significant main effect of drinking history, *F*_(1, 16)_ = 4.556, *p* = 0.0486, also revealed that rats consumed more 10 % ethanol + 25 μg/mL CORT than water regardless of cycles of consumption (Fig. 2C). A significant cycles of consumption × drinking history interaction further clarified this effect, *F*_(1, 16)_ = 16.54, *p* = 0.0009, with *post-hoc* analyses supporting that rats consumed more 10 % ethanol + 25 μg/mL CORT than water in early but not late cycles of consumption (Fig. 2C). Lastly, there was a significant cycles of consumption × sex interaction, *F*_(1, 16)_ = 4.683, *p* = 0.0459, with *post-hocs* uncovering that females consumed more of any solution compared to males in late but not early cycles of consumption (Fig. 2C).

#### 24-hr Ethanol Intake (g/kg).

To evaluate ethanol intake, a mixed 2 × 12 ANOVA with one between-subjects variable (sex) and one repeated measure (cycle) was used. Similar to past experiments, there was a significant main effect of cycle, *F*_(3.6, 28.5)_ = 10.15, *p* < 0.0001, and *post-hoc* analyses revealed that cycles ‘2 versus 9′ and ‘4 versus 7, 10, 11, and 12’ were significantly different (Fig. 2D). There was not a main effect of sex. Data were binned into early (cycles 1–6) versus late (cycles 7–12) cycles of consumption to determine whether the consumption of ethanol was higher in early compared to late cycles of consumption. A mixed 2 × 2 ANOVA with one between-subjects (sex) variable and one repeated variable (cycles of consumption) was used to evaluate sex differences in ethanol consumption in early versus late cycles of consumption. The analysis revealed a significant main effect of cycles of consumption, *F*_(1, 8)_ = 49.86, *p* < 0.0001, with *post-hoc* analyses confirming that rats consumed more ethanol in early versus late cycles of consumption regardless of sex (Fig. 2E). There was no significant main effect of sex.

#### 24-hr CORT Intake (mg/kg).

To evaluate differences in CORT intake between males and females during the drinking paradigm, a mixed 2 × 12 ANOVA with one between-subjects variables (sex) and one repeated measures variable (cycle) was utilized. There was again a significant main effect of cycle, *F*_(3.6, 28.5)_ = 10.15, *p* < 0.0001, and *post-hoc* analyses supported that cycles 2 versus 9; and 4 versus 7, 10–12 were significantly different (Fig. 2F). There was no significant main effect of sex or a sex × cycle interaction. Again, to determine if CORT intake differed in early versus late cycles of the drinking paradigm, a mixed 2 × 2 ANOVA with one between-subjects variable (sex) and one repeated measures (cycles of consumption) was used. There was a significant main effect of cycles of consumption, *F*_(1, 8)_ = 50.07, *p* < 0.0001, such that rats consumed more CORT during early versus late cycles of consumption (Fig. 2G). There was not a significant main effect of sex or a sex × cycles of consumption interaction.

#### Blood-Brain Barrier Permeability effects.

**Fig. 3.**
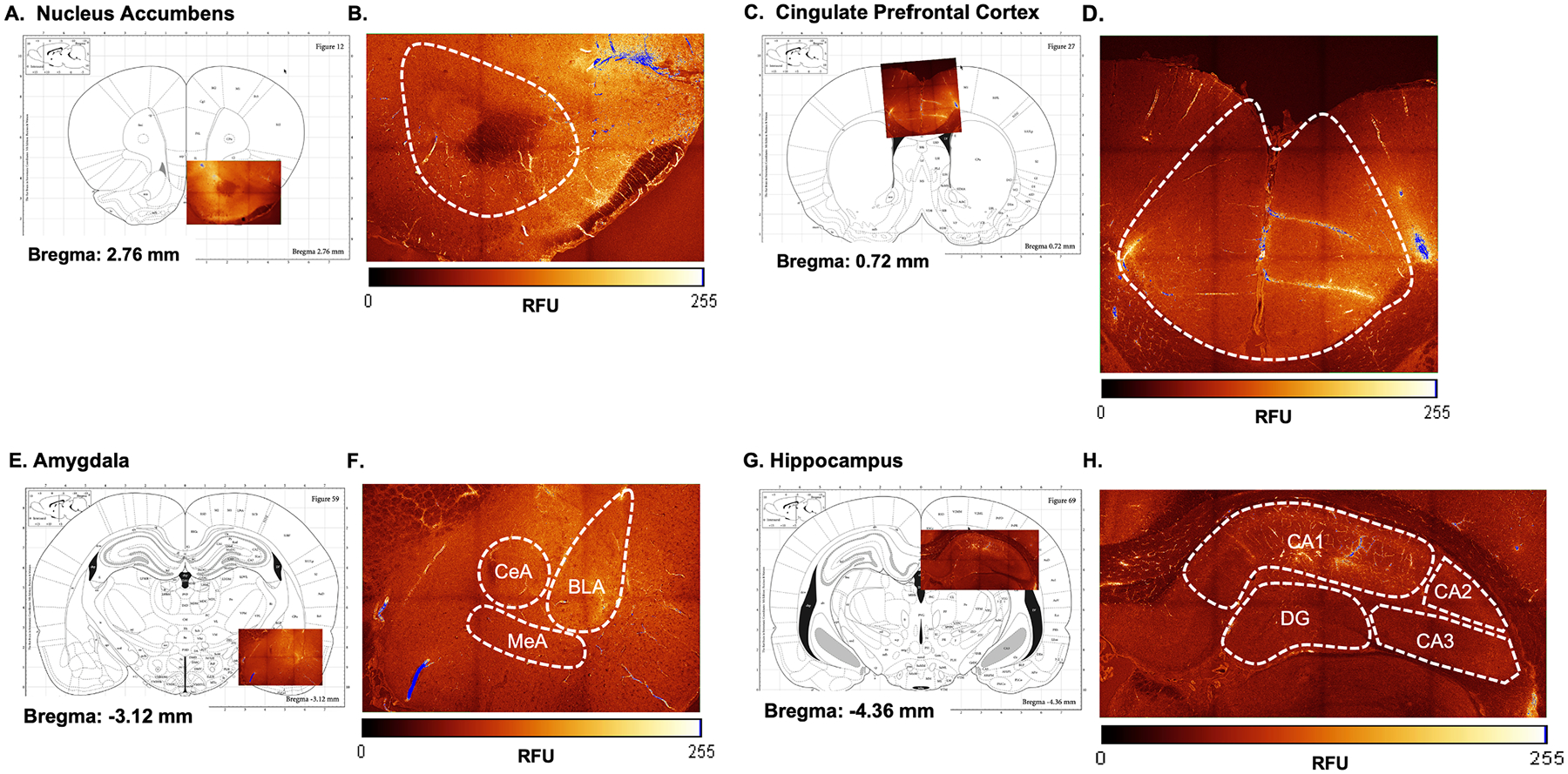
Ethanol and CORT exposure did not affect BBB permeability at the NAc, cPFC, AMG, or HPC. (A) Region coordinates used for the nucleus accumbens and (B) representative image of the region. (C) Region coordinates used for cingulate prefrontal cortex and (D) representative image of the region. (E) Region coordinates used for the amygdala and (F) representative image of the region including the ROI’s of analyzed subregions, basolateral, medial, and central amygdala. (G) Region coordinates used for the hippocampus and (H) representative image of the region including the ROI’s of analyzed subregions, CA1, CA2, CA3, and dentate gyrus.

BBB permeability was evaluated in the cingulate prefrontal cortex (cPFC), nucleus accumbens (NAc), amygdala (AMG), and hippocampus (HPC) because these were the regions in which AIE-induced increases in permeability were found [31]. Images of the amygdala and hippocampus were then subregioned to determine if any differences in permeability existed in the basolateral amygdala (BLA), central amygdala (CeA), or medial amygdala (MeA) or the CA1, CA2, CA3, or dentate gyrus of the HPC. A 2 × 2 factorial ANOVA with two between-subjects variables (sex and drinking history) was conducted to evaluate group differences in permeability. At the NAc, there was a significant main effect of sex, *F*_(1, 36)_ = 4.622, *p* < 0.0384, such that males had increased BBB permeability compared to females (Table 4; for coordinates and representative image see Figs. 3A and 3B). There were no significant differences at the cPFC (Table 4; for coordinates and representative images see Figs. 3C and 3D), whole amygdala or its subregions (Table 4; for coordinates and representative image see Figs. 3E and 3F), or whole hippocampus (Table 4; for coordinates and representative image see Figs. 3G and 3H).

### 3.3. Experiment 3 – adolescent ethanol and CORT effects on ethanol-induced hypothermia

#### Body Weight.

**Fig. 4.**
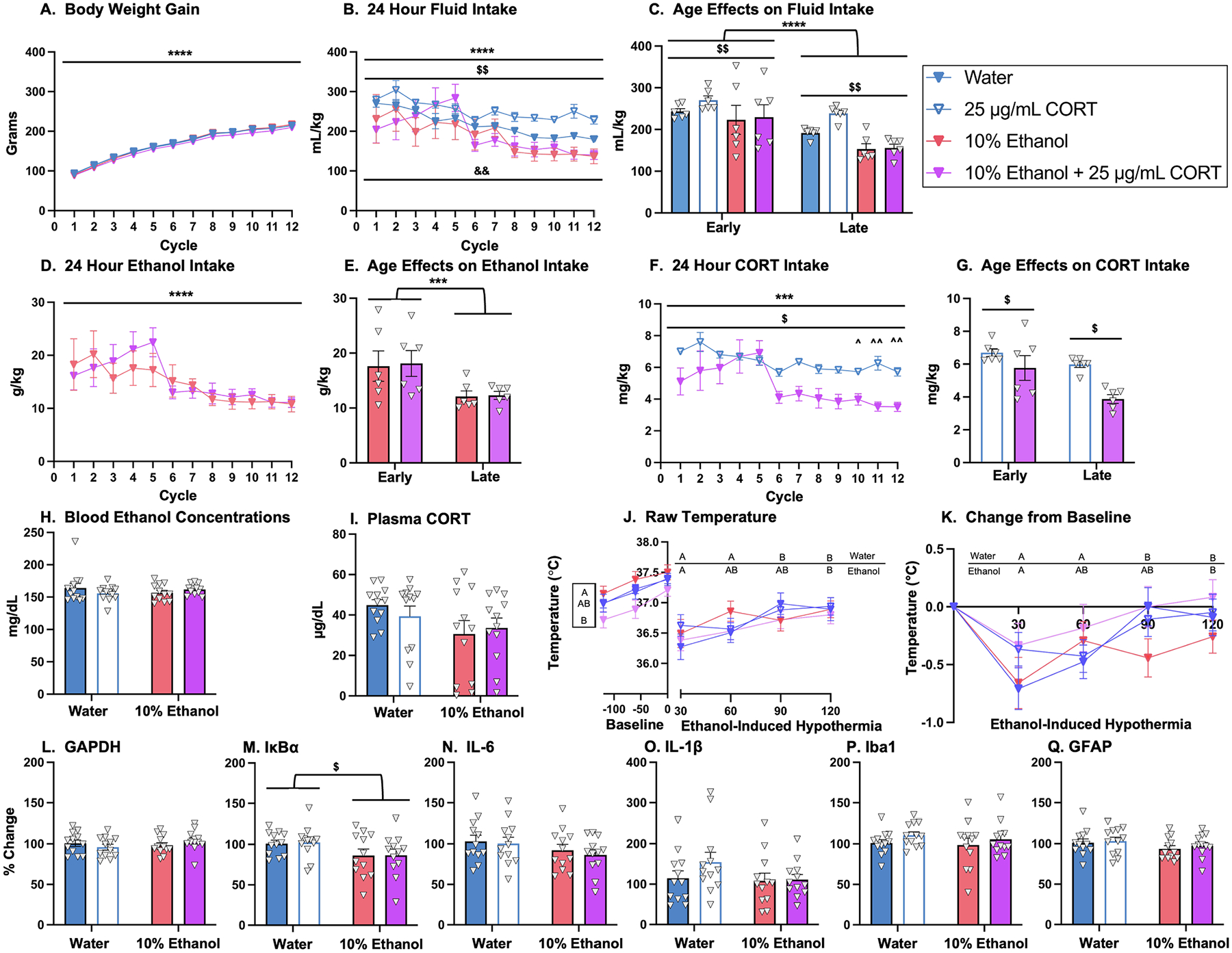
10 % ethanol consumption delayed recovery following ethanol-induced hypothermia and suppressed IκBα mRNA expression. (A) All rats gained weight normally throughout the adolescent drinking paradigm. (B) Fluid consumption (mL/kg) decreased throughout the drinking paradigm for all groups, and 10 % ethanol decreased fluid consumption regardless of CORT or cycle. Rats also consumed more fluid in the presence of 25 μg/mL CORT compared to water alone. (C) Rats consumed more fluids (mL/kg) during the early versus late cycles of consumption. 10 % ethanol also decreased fluid consumption regardless of early versus late cycles. (D) Ethanol consumption (g/kg) decreased throughout the drinking paradigm for all groups. (E) Rats consumed more ethanol (g/kg) during the early versus late cycles of consumption. (F) CORT consumption (mg/kg) decreased throughout the drinking paradigm for all groups. Ethanol also decreased CORT consumption, and this was especially true for cycles 10, 11, and 12. (G) Rats consumed more CORT (mg/kg) in early versus late cycles of consumption, and ethanol decreased CORT consumption. (H) There were no significant differences in BECs which were taken 120 min post-ethanol challenge. (I) There was a trend for ethanol to decrease plasma CORT 120 min post-ethanol challenge. (J) CORT significantly decreased baseline body temperatures. Following ethanol challenge, the raw temperatures of rats with ethanol history recovered more slowly compared to rats with water history. (K) Following ethanol challenge, the change from the first baseline showed that rats with ethanol history recovered more slowly compared to rats with water history. (L) There were no differences in GAPDH mRNA expression which meant it could be used as the housekeeper. (M) Ethanol history significantly decreased IκBα mRNA expression regardless of CORT. (N-Q) No differences in mRNA expression of IL-6, IL-1β, iba1, or gfap were detected. **** indicates main effect of cycle or cycles of consumption, *p* < 0.0001; *** indicates main effect of cycle or cycles of consumption, *p* < 0.001; ** indicates main effect of cycle or cycles of consumption, *p* < 0.001; $$ indicates main effect of ethanol, *p* < 0.01; $ indicates main effect of ethanol, *p* < 0.05; && indicates main effect of CORT, *p* < 0.01; ^^ indicates cycle × ethanol interaction, *p* < 0.01; ^ indicates cycle × ethanol interaction, *p* < 0.05; and letters depict significant *post-hocs* of ethanol × time interaction.

A 2 × 2 × 12 ANOVA with two between-subjects variables (ethanol and CORT) and one repeated measures variable (cycle) was used to evaluate differences in body weight. This analysis revealed a main effect of cycle, *F*_(1.9, 83.3)_ = 3124, *p* < 0.0001, demonstrating that all groups gained weight throughout the drinking procedure (Fig. 4A). There were no other group effects or interactions detected.

#### Total Fluid Intake (mL/kg).

To evaluate differences in fluid intake, a 2 × 2 × 12 mixed-effects model with two between-subjects variables (ethanol and CORT) and one repeated measures variable (cycle) was used. There was a significant main effect of cycle, *F*_(11, 217)_ = 11.62, *p* < 0.0001, and *post-hoc* analyses supported that rats consumed less fluid per body weight as the paradigm progressed (Fig. 4B). A significant main effect of ethanol was also detected, *F*_(1, 20)_ = 10.14, *p* = 0.0047. and *post-hoc* analyses indicated that the presence of ethanol in the drinking solution decreased fluid intake in comparison to groups that did not consume ethanol (Fig. 4B). To determine if water intake was increased by 25 μg/mL, a 2 × 12 mixed-effects model with one between-subjects variable (CORT) and one repeated measures variable (cycle) was used. There was a significant main effect of cycle, *F*_(4.2, 41.6)_ = 17.60, *p* < 0.0001, which supported that fluid intake decreased as the drinking paradigm progressed (Fig. 4B). There was also a significant main effect of CORT, *F*_(1, 10)_ = 16.61, *p* = 0.0022, and *post-hoc* analyses supported that 25 μg/mL significantly increased consumption compared to water alone throughout the drinking paradigm (Fig. 4B). Similar to Experiments 1 and 2, data were binned into early (cycles 1–6) versus late (cycles 7–12) cycles of consumption. A 2 × 2 × 2 mixed ANOVA with two between-subjects variables (ethanol and CORT) and one repeated measure (cycles of consumption) was used to analyze these data. Again, there was a significant main effect of cycles of consumption, *F*_(1, 20)_ = 28.12, *p* < 0.0001, determining that rats consumed more mL/kg during early compared to late cycles (Fig. 4C). There was also a significant main effect of ethanol, *F*_(1, 20)_ = 9.958, *p* = 0.0050, supporting that ethanol-consuming rats consumed less fluid during both early and late cycles (Fig. 4C).

#### 24-hr Ethanol Intake (g/kg).

To evaluate differences in ethanol intake (g/kg), a 2 × 12 mixed-effects model with one between-subjects variables (CORT) and one repeated measures variable (cycle) was used. There was a significant main effect of cycle, *F*_(11, 108)_ = 5.543, *p* < 0.0001, which indicated that rats decreased their ethanol intake as the drinking model progressed (Fig. 4D). Data were binned into early (cycles 1–6) versus late (cycles 7–12) cycles, and a 2 × 2 mixed ANOVA with one between-subjects variables (CORT) and one repeated measure (cycles of consumption) determined that there was a significant main effect of cycles of consumption, *F*_(1, 10)_ = 12.06, *p* = 0.0060, further supporting that rats consumed more ethanol during early versus late cycles of consumption (Fig. 4E).

#### 24-hr CORT Intake (mg/kg).

To assess differences in CORT intake (mg/kg), a 2 × 12 mixed-effects model with one between-subjects variables (ethanol) and one repeated measures variable (cycle) was used. There was a significant main effect of cycle, *F*_(3.3, 32.2)_ = 7.820, *p* = 0.0004, which indicated that rats decreased their CORT intake as the drinking model progressed (Fig. 4F). There was also a significant main effect of ethanol, *F*_(1, 10)_ = 8.282, *p* = 0.0164, and *post-hoc* analyses supported that ethanol decreased CORT intake (Fig. 4F). There was a significant ethanol × cycle interaction, *F*_(11, 109)_ = 2.413, *p* = 0.0100, and *post-hoc* analyses indicated that ethanol decreased CORT intake during cycles 10, 11, and 12, but not within any of the other cycles (Fig. 4F). Data were binned into early (cycles 1–6) versus late (cycles 7–12) cycles, and a 2 × 2 mixed ANOVA with one between-subjects variables (ethanol) and one repeated measure (cycles of consumption) determined that there was a significant main effect of cycles of consumption, *F*_(1, 10)_ = 19.53, *p* = 0.0013, further supporting that rats consumed more CORT during early versus late cycles of consumption (Fig. 4G). The main effect of ethanol was significant, *F*_(1, 10)_ = 8.34, *p* = 0.0162, indicating that ethanol decreased CORT intake (Fig. 4G).

#### Blood Ethanol Concentrations (BECs).

BECs were analyzed to determine if there was any groups differences 120-min post 2.0 g/kg ethanol challenge. A 2 × 2 ANOVA with two between-subjects variables (ethanol and CORT) was used. There were no significant main effects or an interaction between the variables (Fig. 4H).

#### Plasma Corticosterone (CORT).

Plasma CORT was analyzed to determine if there were any group differences in CORT 120-min post 2.0 g/kg ethanol challenge. A 2 × 2 ANOVA with two between-subjects variables (ethanol and CORT) was used. Although it did not achieve statistical significance, there was a trend for a main effect of ethanol, *F*_(1, 44)_ = 3.974, *p* = 0.0524, indicating that history 10 % ethanol decreased plasma CORT 120-min post 2.0 g/kg ethanol challenge (Fig. 4I).

#### Ethanol-Induced Hypothermia.

To determine if there were any group differences in baseline temperatures prior to the ethanol challenge, a 2 × 2 × 3 mixed ANOVA with two between-subjects variables (ethanol and CORT) and one repeated measures variable (baseline temperature) was used. A significant main effect of CORT was detected, *F*_(1, 44)_ = 4.363, *p* = 0.0425, which indicated that CORT decreased the baseline temperatures (Fig. 4J). The trending ethanol × CORT interaction *F*_(2, 88)_ = 0.7415, *p* = 0.0512 suggested that the main effect of CORT was primarily driven by the group that consumed 25 μg/mL CORT but was rescued by the addition of 10 % ethanol (Fig. 4J) however, this effect did not achieve statistical significance. Following the ethanol challenge, core body temperatures were measured to assess ethanol-induced hypothermia. The raw temperatures were assessed using a 2 × 2 × 4 mixed ANOVA with two between-subjects variables (ethanol and CORT) and one repeated measures variable (timepoint; 30-, 60-, 90-, 120-min post-injection). There was a significant main effect of timepoint, *F*_(3, 132)_ = 19.38, *p* < 0.0001, indicating that temperatures increased across the timepoints as rats returned to baseline temperatures (Fig. 4J). There was also a significant timepoint × ethanol interaction, *F*_(3, 132)_ = 2.969, *p* = 0.0343, and *post-hoc* analyses indicated that rats with a water history recovered quicker following the ethanol challenge (90-min timepoint) compared to rats with an ethanol history (120-min timepoint; Fig. 4J).

To examine differences in ethanol-induced hypothermia and account for baseline differences, analyses were completed on change from the first baseline. The first baseline was used rather than the later baselines because it had the least contamination from the presence of the researcher and stress of injection and handling. A 2 × 2 × 4 mixed ANOVA with two between-subjects variables (ethanol and CORT) and one repeated measures variable (timepoint; 30-, 60-, 90-, 120-min post-injection) was used. There was a significant main effect of timepoint, *F*_(3, 132)_ = 19.38, *p* < 0.0001, indicating that body temperature increased following the injection for rats of all groups (Fig. 4K). Interestingly, the ethanol × timepoint interaction was significant, *F*_(3, 132)_ = 2.969, *p* = 0.0343. *Post-hoc* analyses found that the water groups returned to baseline at 90-min post-injection while the ethanol groups did not return to baseline until 120-min post-injection regardless of CORT exposure (Fig. 4K).

### Gene Expression in the Hippocampus.

RT-PCR was performed on brain punches of the HPC to evaluate changes in mRNA expression following intermittent ethanol and CORT intake followed by ethanol challenge. A 2 × 2 ANOVA with two between-subjects variables (ethanol and CORT) revealed no significant differences in housekeeper gene expression, GAPDH, which allowed for the normalization of the following PCR data to the respective housekeeper expression of each rat (Fig. 4L). A significant main effect of ethanol was noted in IκBα mRNA expression, *F*_(1, 44)_ = 5.512, *p* = 0.0234, with rats with an ethanol history showing decreased IκBα levels relative to comparators (Fig. 4M). For IL-6, IL-1β, Iba1, and GFAP, no significant differences were detected (Figs. 4N–Q).

### 3.4. Experiment 4 – adolescent ethanol and CORT effects on Poly I:C-induced fever

#### Body Weight.

**Fig. 5.**
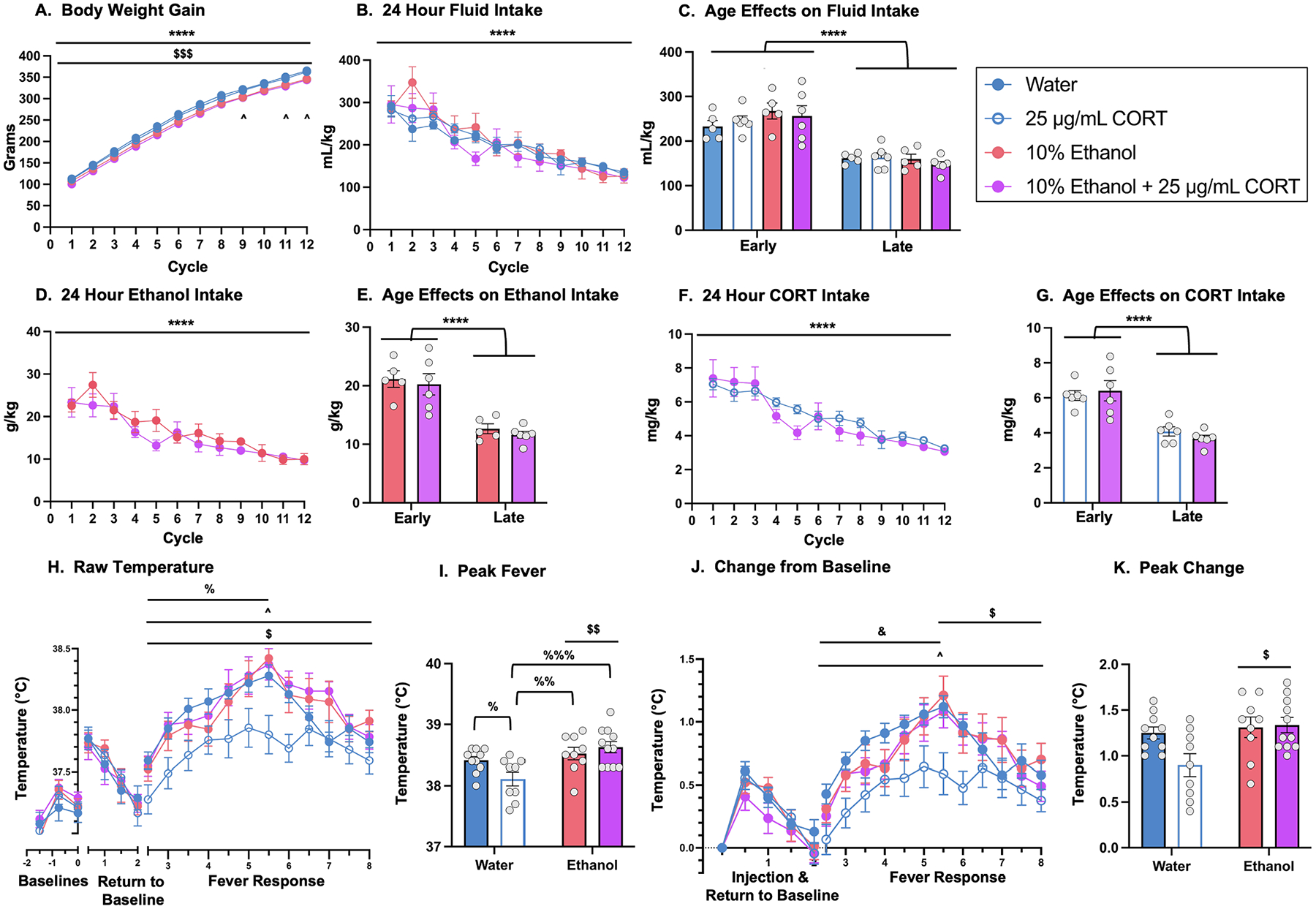
25 μg/mL CORT consumption suppressed while 10 % ethanol sensitized Poly I:C-induced fever. (A) All rats gained weight throughout the adolescent drinking paradigm. Ethanol decreased body weight, and this was especially true for cycles 9, 11, and 12. (B) Fluid consumption (mL/kg) decreased throughout the drinking paradigm for all groups. (C) Rats consumed more fluids (mL/kg) during the early versus late cycles of consumption. (D) Ethanol consumption (g/kg) decreased throughout the drinking paradigm for all groups. (E) Rats consumed more ethanol (g/kg) during the early versus late cycles of consumption. (F) CORT consumption (mg/kg) decreased throughout the drinking paradigm for all groups. (G) Rats consumed more CORT (mg/kg) in early versus late cycles of consumption. (H) In raw body temperatures, there were no differences in baseline temperatures or the return to baseline. Following Poly I:C challenge, rats mounted a fever response, and history of ethanol sensitized the fever response. (I) Ethanol increased the peak fever, and rats with history of 25 μg/mL CORT had the lowest peak fever compared to all other groups. (J) In change from the first baseline, there were no differences in the return to baseline following Poly I:C injection. Rats mounted a fever response, and CORT suppressed the rise in temperature following the challenge. During the fall in temperature, ethanol history significantly increased body temperature. (K) History of ethanol increased the peak change from baseline. There was also a trend in which the 25 μg/mL CORT had the lowest peak change compared to all other groups. **** indicates main effect of cycle or cycles of consumption, *p* < 0.0001; $$$ indicates main effect of ethanol, *p* < 0.001; $$ indicates main effect of ethanol, *p* < 0.01; $ indicates main effect of ethanol, *p* < 0.05; ^ indicates cycle × ethanol interaction, *p* < 0.05; & indicates main effect of CORT, *p* < 0.05; % % % indicates ethanol × CORT interaction, *p* < 0.001; % % indicates ethanol × CORT interaction, *p* < 0.01; % indicates ethanol × CORT interaction, *p* < 0.05.

A 2 × 2 × 12 mixed ANOVA with two between-subjects variables (ethanol and CORT) and one repeated measures variable (cycle) was used to evaluate differences in body weight. This analysis revealed a significant main effect of cycle, *F*_(1.4, 54.9)_ = 6367, *p* < 0.0001, demonstrating that all groups gained weight throughout the drinking procedure (Fig. 5A). In addition, there was a significant main effect of ethanol, *F*_(1, 40)_ = 13.34, *p* = 0.0007, and *post-hocs* indicated that rats that consumed ethanol weighed significantly less regardless of cycle or CORT exposure (Fig. 5A). The analyses also uncovered a significant cycle × ethanol interaction, *F*_(11, 440)_ = 2.577, *p* = 0.0035, which indicated that rats that consumed 10 % ethanol weighed significantly less than rats that consumed water at cycles 9, 11, and 12 (Fig. 5A).

#### Total Fluid Intake (mL/kg).

To evaluate differences in fluid intake, a 2 × 2 × 12 mixed-effects model with two between-subjects variables (ethanol and CORT) and one repeated measures variable (cycle) was used. As expected, there was a significant main effect of cycle, *F*_(4.7, 80)_ = 37.77, *p* < 0.0001, and *post-hoc* analyses supported that rats consumed less fluid per body weight as the cycles of consumption continued (Fig. 5B). Similar to earlier experiments, data were binned into early (cycles 1–6) versus late (cycles 7–12) cycles of consumption. A 2 × 2 × 2 mixed ANOVA with two between-subjects variables (ethanol and CORT) and one repeated measure (cycles of consumption) was used to analyze these data. Again, there was a significant main effect of cycles of consumption, *F*_(1, 18)_ = 132.0, *p* < 0.0001, which indicated that rats consumed more mL/kg during early compared to late cycles (Fig. 5C).

#### 24-hr Ethanol Intake (g/kg).

To evaluate differences in ethanol intake (g/kg), a 2 × 12 mixed-effects model with one between-subjects variables (CORT) and one repeated measures variable (cycle) was used. There was a significant main effect of cycle, *F*_(4.4, 35.7)_ = 15.43, *p* < 0.0001, which indicated that rats decreased their ethanol intake as the drinking model progressed (Fig. 5D). There were no other significant main effects or interactions. Data were binned into early (cycles 1–6) versus late (cycles 7–12) cycles, and a 2 × 2 mixed ANOVA with one between-subjects variables (CORT) and one repeated measure (cycles of consumption) determined that there was a significant main effect of cycles of consumption, *F*_(1, 9)_ = 63.83, *p* < 0.0001, further supporting that rats consumed more ethanol during early versus late cycles of consumption (Fig. 5E).

#### 24-hr CORT Intake (mg/kg).

To assess differences in CORT intake (mg/kg), a 2 × 12 mixed-effects model with one between-subjects variables (ethanol) and one repeated measures variable (cycle) was used. There was a significant main effect of cycle, *F*_(3.3, 32.4)_ = 19.41, *p* < 0.0001, which indicated that rats decreased their CORT intake as cycles of the drinking model continued (Fig. 5F). Data were binned into early (cycles 1–6) versus late (cycles 7–12) cycles, and a 2 × 2 mixed ANOVA with one between-subjects variables (ethanol) and one repeated measure (cycles of consumption) determined that there was a significant main effect of cycles of consumption, *F*_(1, 10)_ = 59.98, *p* < 0.0001, further supporting that rats consumed more ethanol during early versus late cycles of consumption (Fig. 5G).

#### Poly I:C fever.

To determine if there were any group differences in baseline temperatures prior to the Poly I:C challenge, a 2 × 2 × 3 mixed ANOVA with two between-subjects variables (ethanol and CORT) and one repeated measures variable (baseline temperature) was used. A significant main effect of baseline temperatures was detected, *F*_(1.2, 41.8)_ = 26.58, *p* < 0.0001, which indicated that baseline temperatures increased as time progressed likely due to the presence of the researcher (Fig. 5H). To evaluate if there were any group differences in core body temperatures during the return to baseline post-injection, a 2 × 2 × 5 mixed ANOVA with two between-subjects variables (ethanol and CORT) and one repeated measures variable (timepoint: 0–2 hr) was used. There was a significant main effect of timepoint, *F*_(3.710, 129.9)_ = 48.34, *p* < 0.0001, which indicated that body temperature decreased, and importantly, there were no group differences or interactions (Fig. 5H). To evaluate differences in the overall fever response, a 2 × 2 × 13 mixed ANOVA with two between-subjects variables (ethanol and CORT) and one repeated measures variable (timepoint: 2–8) was used. There was a significant main effect of timepoint, *F*_(6, 208.6)_ = 40.95, *p* < 0.0001, which supported that rats mounted a fever because their temperatures increased (Fig. 5H). There was also a significant main effect ethanol, *F*_(1, 35)_ = 4.212, *p* = 0.0477, and *post-hoc* analyses concluded that rats with ethanol history had significantly higher core body temperatures during the Poly I:C-induced fever (Fig. 5H). There was also a significant timepoint × ethanol interaction, *F*_(12, 420)_ = 1.973, *p* = 0.0252, but *post-hoc* analyses did not find any timepoints at which rats with an ethanol history has significantly different body temperatures than rats with a water history (Fig. 5H). To further analyze changes in Poly I:C-induced fever, the rise in core body temperature was analyzed separately using a 2 × 2 × 8 mixed ANOVA with two between-subjects variables (ethanol and CORT) and one repeated measures variable (timepoint: 2–5.5 hr). There was a significant main effect of timepoint, *F*_(3.9, 136.6)_ = 85.93, *p* < 0.0001, which supported that core body temperatures increased during the Poly I:C-induced fever rise (Fig. 5H). There was also a significant time × ethanol interaction, *F*_(7, 245)_ = 2.869, *p* = 0.0068, but *post-hoc* analyses did not find any timepoints at which rats with an ethanol history has significantly different body temperatures than rats with a water history (Fig. 5H). The ethanol × CORT interaction was also significant, *F*_(1, 35)_ = 4.202, *p* = 0.0479. There were no significant *post-hoc* analyses, but the 25 μg/mL CORT group was nearly significantly different from rats that solely consumed water (*p* = 0.0730) and rats that consumed 10 % ethanol + 25 μg/mL CORT (*p* = 0.0773; Fig. 5H). The fall in core body temperature post-peak fever was also evaluated separately using a 2 × 2 × 6 mixed ANOVA with two between-subjects variables (ethanol and CORT) and one repeated measures variable (timepoint: 5.5–8 hr). A significant main effect of timepoint was detected, *F*_(3.6, 126.3)_ = 15.20, *p* < 0.001, indicating that core body temperature of all rats decreased (Fig. 5H). There was also a significant main effect of ethanol, *F*_(1, 35)_ = 7.737, *p* = 0.0087, and *post-hoc* analyses confirmed that history of ethanol increased core body temperatures compared to rats with a history of water consumption (Fig. 5H). Lastly, peak fever (highest raw temperatures per individual animal) was evaluated using a 2 × 2 ANOVA with two between-subjects variables (ethanol and CORT). There was a significant main effect of ethanol, *F*_(1, 35)_ = 11.32, *p* = 0.0019, with rats that consumed ethanol having an increased peak fever compared to rats that did not consume ethanol (Fig. 5I). There was also a significant ethanol × CORT interaction, *F*_(1, 35)_ = 4.678, *p* = 0.0375, and *post-hoc* analyses indicated that the 25 μg/mL CORT group had a peak temperature that was significantly lower than all other groups (Fig. 5I).

Although there were no significant group differences in baseline temperatures, change from baseline was calculated to account for subtle individual differences in baseline body temperature. The first baseline was used to calculate change from baseline because it was the least contaminated by the presence of the researcher indicated by the increase in baseline temperatures (main effect of baseline temperatures). A 2 × 2 × 5 mixed ANOVA with two between-subjects variables (ethanol and CORT) and one repeated measures variable (timepoint: 0–2 hr) was used to determine whether there were differences in return to baseline post-injection. There was a significant main effect of timepoint, *F*_(3.6, 125.1)_ = 64.86, *p* < 0.0001, which indicated that core body temperatures decreased over time, but importantly, there were no group differences (Fig. 5J). To evaluate differences in the fever response, a 2 × 2 × 13 mixed ANOVA with two between-subjects variables (ethanol and CORT) and one repeated measures variable (timepoint: 2–8) was used. There was a significant main effect of timepoint, *F*_(6, 208.6)_ = 40.95, *p* < 0.0001, which indicated that core body temperatures changed throughout the fever response regardless of group (Fig. 5J). The timepoint × ethanol interaction was also significant, *F*_(12, 420)_ = 1.973, *p* = 0.0252, but *post-hoc* analyses did not find any timepoints at which rats with an ethanol history has significantly different body temperatures than rats with a water history. To further analyze changes in Poly I:C-induced fever, the rise in core body temperature was analyzed separately using a 2 × 2 × 8 mixed ANOVA with two between-subjects variables (ethanol and CORT) and one repeated measures variable (timepoint: 2–5.5 hr). Again, there was a significant main effect of timepoint, *F*_(3.9, 136.6)_ = 85.93, *p* < 0.0001 (Fig. 5J). There was also a significant main effect of CORT, *F*_(1, 35)_ = 4.478, *p* = 0.0415, and *post-hoc* analyses concluded that CORT significantly decreased core body temperature during the fever-induced rise in body temperature (Fig. 5J). In addition, the timepoint × ethanol interaction was significant, *F*_(7, 245)_ = 3.869, *p* = 0.0068, but *post-hoc* analyses did not find any timepoints at which rats with an ethanol history has significantly different body temperatures than rats with a water history. The fall in core body temperature post-peak fever was also evaluated separately using a 2 × 2 × 6 mixed ANOVA with two between-subjects variables (ethanol and CORT) and one repeated measures variable (timepoint: 5.5–8 hr). There was a significant main effect of timepoint, *F*_(3.6, 126.3)_ = 15.20, *p* < 0.0001, because core body temperature significantly decreased regardless of group (Fig. 5J). There was also a significant main effect of ethanol, *F*_(1, 35)_ = 5.863, *p* = 0.0208, which indicated that core body temperatures of rats with ethanol history were higher compared to rats that consumed ethanol regardless of timepoint and CORT (Fig. 5J). Lastly, peak change in temperature (largest change from initial baseline for each rat) was evaluated using a 2 × 2 ANOVA with two between-subjects variables (ethanol and CORT). A significant main effect of ethanol was detected, *F*_(1, 35)_ = 6.679, *p* = 0.0141, which depicted that ethanol increased peak change in temperature (Fig. 5K). The ethanol × CORT interaction nearly achieved significance, *F*_(1, 35)_ = 3.800, *p* = 0.0593, and *post-hoc* analyses indicated that the 25 μg/mL CORT group had a peak change in temperature that was significantly lower than all other groups (Fig. 5K). This supported that the main effect of ethanol was likely driven by the low peak change in temperature of the 25 μg/mL CORT group.

## 4. Discussion

Past studies have concluded that long-term exposure to CORT in the drinking water increased ethanol consumption in adrenalectomized rats [13], increased ethanol self-administration in male rats [14], and augmented cue-induced reinstatement of ethanol-seeking in in adolescent-exposed female rats [15]. The current experiments first evaluated the concentration-dependent effects of CORT on ethanol intake (Exp1). It was hypothesized *a priori* that the lowest concentration of CORT (25 μg/mL) would increase consumption of 10 % ethanol. This was supported by Exp 1 because rats consumed more 10 % ethanol when it was co-consumed with 25 μg/mL CORT. However, there was no effect of group when all concentrations of CORT were analyzed, meaning there was not a robust effect of CORT on 10 % ethanol consumption, or it may be concentration-specific due to inactivation of the HPA axis [54] at the higher concentrations. In Exp 2, rats that consumed 10 % ethanol + 25 μg/mL CORT consumed significantly more fluid (mL/kg) compared to rats that consumed water, an effect that was true for males and females. However, in Experiments 3 and 4, 25 μg/mL CORT did not increase ethanol consumption in females (Exp 3) or males (Exp 4). In fact, the results of Experiment 3 supported that 25 μg/mL CORT increased consumption of water but not 10 % ethanol. Overall, the effects of 25 μg/mL CORT on intake were variable in how or whether they presented across studies, and it appears that 25 μg/mL CORT may variably affect general fluid consumption, not as an ethanol-specific effect. This is consistent with prior studies showing that the influence of stress exposure on ethanol intake is inconsistent across published studies, and likely depends on the species used, the nature of the stress challenge imposed, timing of the stress-ethanol exposures, and other features of the experiments [55].

Chronic stress has been shown to have long-lasting effects on the neuroimmune system, producing sensitized microglial activation [17], as well as changes in peripheral organ weights such as the thymus, adrenals, and spleen [38]. Indeed, our recent work showed that acute versus chronic stress had a differential impact on ethanol-induced neuroimmune gene expression ([56]; see [57] for a recent review). The current experiments examined restraint-evoked changes in neuroimmune gene expression and CORT reactivity following intermittent exposure to 10 % ethanol with or without CORT (Exp 1), which has previously been shown to elicit a moderate increase in IL-1β expression in the PVN [58]. No long-lasting effects measured by organ weights (thymus, adrenals, spleen) or peak CORT release were detected following the drinking paradigm, while there was a small increase in mRNA gene expression of IL-6 in the HPC in 10 % ethanol + 100 μg/mL CORT compared to water controls. This is supported by Fitzwater [56] which found that rats that underwent footshock and ethanol challenge had higher IL-6 mRNA expression compared to rats that underwent the ethanol challenge alone, supporting that IL-6 may be especially sensitive to the interactive effects of stress/chronic CORT and ethanol. Gourley et al. [8] found that adrenal gland weights are unchanged two weeks after CORT consumption, indicating that the adrenals recover rapidly from chronic CORT consumption. One study also compared acute stress paradigms, restraint stress, foot shock, and forced swim, and found that each stressor elevated plasma CORT levels, but 30 min of foot shock or forced swim resulted in significantly higher plasma CORT levels compared to 30 min of restraint [58]. Restraint stress also presents within- and between-session habituation [38]. In the present study, all rats were administered 30 min of restraint stress, which means we cannot determine whether restraint altered neuroimmune gene expression relative to non-stressed controls. This was a known limitation in the study design, but it does not prevent a comparison of whether neuroimmune gene expression in restrained rats was modifiable by a history of CORT and ethanol consumption. In addition, Experiment 3 evaluated plasma CORT 120-min post ethanol challenge, in which there was a trend for history of 10 % ethanol to decrease CORT. Although a full characterization of the CORT response would be needed, this may indicate that history of 10 % ethanol altered HPA axis shutoff or the trajectory of CORT release. However, it remains unknown whether the concurrent 10 % ethanol and CORT model influence diurnal variations in HPA axis function, and this is a logical next step for the current model.

Independently, both chronic stress [20,28,29] and alcohol exposure [21,30,31] have been shown to increase BBB permeability, another aspect of the neuroimmune system. Therefore, the current experiments determined whether the co-consumption of 10 % ethanol + 25 μg/mL CORT affected BBB permeability in males or females, and it was hypothesized that the synergistic effects of ethanol and CORT would cause increased BBB permeability. Here, no differences in BBB permeability were observed due to ethanol and CORT exposure at the NAc, cPFC, AMG, HPC, or various subregions of the AMG (BLA, MeA, and CeA) or HPC (CA1, CA2, CA3, DG). A small change in BBB permeability was detected between males and females in the nucleus accumbens, supporting that the current measures of BBB permeability were sensitive enough to detect changes. We recently showed that adolescent intermittent ethanol exposure increased BBB permeability in males [31] after gavage administration of 4.0 g/kg ethanol which results in BECs of about 175–225 mg/dl [59]. However, the drinking procedure used here produces more moderate BECs in the range of about range of 40–100 mg/dl [33]. Therefore, this may indicate that extreme binge-level BECs are required to influence BBB permeability. Similarly, past studies that support stress-induced increased BBB permeability utilized chronic stress exposures which influence expression of upstream factors of HPA axis activation such as CRH and ACTH, factors that may not be expressed in chronic CORT exposure. Therefore, consumption of 10 % ethanol + 25 μg/mL CORT may have no effect on BBB permeability because the resulting BECs are moderate (40–100 mg/dl), and changes in CRH and ACTH expression may be required.

It is known that exposure to high doses of ethanol cause a rapid decrease in core body temperature known as ethanol-induced hypothermia [48,50]. Following chronic ethanol exposure, one study found that rats displayed a trend towards less hypothermia [60]. Another study using female mice found that chronic ethanol caused a less robust hypothermic response compared to their ethanol-naïve counterparts, only on the first day of ethanol-induced hypothermia [61]. However, some data support that female rats chronically given an ethanol liquid diet had a more robust hypothermic response compared to ethanol-naïve females [50]. In Experiment 3, female rats with a history of ethanol presented a delayed recovery following the adult ethanol challenge. A recent publication from our laboratory found that AIE increased IκBα mRNA expression [62]. Interestingly, in the current study, rats with an ethanol history had decreased IκBα mRNA expression which may indicate that their neuroimmune system was less reactive, potentially contributing to their delayed recovery. Past studies have primarily focused on binge-level ethanol and dependence, so it is possible that moderate ethanol consumption does not have the same effect on the neuroimmune system or ethanol-induced hypothermia.

A recent publication from our laboratory also found that adolescent intermittent ethanol (AIE) via gavage caused a male-specific sensitized Poly I:C fever in adulthood [26], but it was unknown whether this effect would translate to moderate ethanol exposure during adolescence. In addition, studies have supported a variable relationship between CORT and fever depending on the timing of administration. For example, when CORT was administered 24 h before lipopolysaccharide (LPS; bacterial mimetic), a potentiated fever and sickness behavior occurred [63]. However, when CORT was administered one hour prior, it blocked the IL-1β- [64] and LPS-induced fever [65]. In both cases, adrenalectomy completely abolished the fever response to IL-1β [64] or LPS [65]. Although studies have not evaluated fever following chronic CORT exposure, the availability of CORT has the capacity to modulate fever. In Experiment 4, both 10 % ethanol and 25 μg/mL CORT influenced Poly I: C-induced fever. Specifically, 10 % ethanol sensitized the fever response in both the rise and fall of the Poly I:C-induced fever. However, this main effect of ethanol was likely driven by the robust suppression that 25 μg/mL CORT caused. History of 25 μg/mL CORT suppressed the rise in Poly I:C fever, an effect which was rescued in animals that consumed 10 % ethanol + 25 μg/mL CORT, supporting that there are not synergistic effects between ethanol and CORT, and that there may be more complex interactions occurring during prolonged exposure.

### 4.1. Conclusions and future directions

In adolescence, stress and alcohol exposure routinely occur together, and are arguably a hallmark of the developmental period [3], but many publications that utilized rodent models evaluate these exposures separately. The present model of concurrent consumption of ethanol and CORT provides a novel and highly tractable model for concurrent CORT exposure and alcohol consumption, and the studies provided here exude excellent benchmarks for evaluation of this model compared to other strategies used by our lab and others, often including gavage and exceedingly high concentrations of ethanol or CORT. In past studies of ethanol consumption, adolescents were found to consume more than adults [33,66]. In the current experiments, male and female rats consumed more fluids (water and 10 % ethanol +/− CORT) during early adolescence compared to later cycles of consumption, which was also true for the “on” and “off” consumption days of the drinking model. Past studies that utilized CORT consumption also found that mice and rats were modestly lighter compared to controls when they consumed 25 μg/mL (mice) and 50 μg/mL (rat) CORT [8], an effect that resolved when CORT consumption was terminated. In Experiment 1, the highest concentration of CORT (100 μg/mL) modestly suppressed weight gain.

The current studies found minimal long-lasting effects in neuroimmune sensitization following restraint stress, changes in organ weights, or alteration in BBB permeability. However, there were many effects in neuroimmune reactivity that emerged following an adult challenge of either ethanol or Poly I:C, suggesting that the impact of concurrent ethanol and CORT consumption may remain latent until later provocation. The diversity in lasting effects to the neuroimmune system may be because the current drinking model produces moderate BEC’s, and utilizes chronic CORT exposure, which is just one element of overall HPA axis activation. Therefore, the long-term effects to the CNS may be more moderate. For this reason, the concurrent consumption model may be used to delineate effects of moderate ethanol and CORT exposure (more comparable to the vast majority of individual’s adolescent experience) rather than high-intensity binge ethanol and stress exposure which are often the target of preclinical studies. Indeed, the relatively modest effects of the model on gross measures of growth (weight gain) and organ weights suggest this model is well tolerated and may provide a path towards understanding neurobehavioral effects of concurrent CORT and ethanol that are independent of other, off-target physiological effects. Though the concurrent drinking model used here produced minimal effects on ethanol intake, this is consistent with a wide body of literature showing that stress effects on ethanol intake are highly variable and reflect differences in stress model, strain, timing, and other features of the experimental design [55]. Thus, the present model of concurrent ethanol and CORT consumption may provide a tractable approach for evaluation of moderate ethanol effects and how they interact with the principal hormone of the HPA axis.

## Figures and Tables

**Table 1:** Forward and reverse primer sequence of primers used in RT-PCR.

Primer	Accession Number	Primer Sequence (5′ to 3′)	
		Forward Primer	Reverse Primer
GAPDH	NM_017008	GTGCCAGCCTCGTCTCATAG	AGAGAAGGCAGCCCTGGTAA
IL-1β	NM_031512.2	TCCTCTGTGACTCGTGGGAT	TGGAGAATACCACTTGTTGGCT
IL-6	NM_012589.2	TAGTCCTTCCTACCCCAACTTCC	TTGGTCCTTAGCCACTCCTTC
IκBα	NM_001105720.2	CTGTTGAAGTGTGGGGCTGA	AGGGCAACTCATCTTCCGTG
Iba1	NM_017196.3	TCATCGTCATCTCCCCACCTA	GGGCTTTCAGCAGTCCAAAAG
GFAP	NM_017009.2	GGCGAAGAAAACCGCATCA	CTCCACCGTCTTTACCACGA
GR	NM_012576.1	GGTTAGCAGAGGGAGGCTTT	AGGGTGGGGAGGATTAGTGT
MR	NM_013131.1	TAGTCGGTCTGGGATTTTGC	TCAGGCTTCCTTGTTGGTTC

Primer name, accession number, and forward and reverse primer sequences from 5′ to 3′. These primers were used to evaluate mRNA expression in RT-PCR in Exp 1 and Exp 3.

**Table 2:** Plasma CORT and organ weights following 30 min of restraint or ethanol challenge.

Adolescent History	Water	10 % Ethanol	10 % Ethanol + 25 μg/mL CORT	10 % Ethanol + 50 μg/mL CORT	10 % Ethanol + 100 μg/mL CORT
Peak Plasma Corticosterone (μg/dL)					
CORT	49.5 ± 3.7	53.8 ± 2.6	51.3 ± 1.9	51.0 ± 3.0	52.3 ± 3.5
Organ weight (mg)					
Adrenal Glands	75.4 ± 4.5	80.8 ± 4.8	68.7 ± 3.5	77.9 ± 2.4	72.7 ± 2.6
Spleen	631.2 ± 15.9	652.0 ± 20.5	612.7 ± 29.4	597.9 ± 12.4	578.6 ± 31.2
Thymus	351.4 ± 13.2	368.3 ± 21.8	347.1 ± 26.9	351.8 ± 14.4	345.5 ± 20.3
Organ weight adjusted to body weight (mg/kg)					
Adrenal Glands	303.4 ± 20.6	311.5 ± 18.8	279.0 ± 13.2	320.2 ± 12.2	304.5 ± 11.4
Spleen	2527 ± 54.0	2510 ± 73.3	2473 ± 66.0	2449 ± 37.4	2411 ± 101.7
Thymus	1407 ± 50.3	1427 ± 96.1	1394 ± 84.9	1440 ± 53.2	1451 ± 94.3

In Exp 1, immediately following 30 min of restraint stress, tissue was collected, and organ weights were recorded to assess plasma corticosterone (μg/dL); weights of adrenal glands, spleen, and thymus (mg); and the same organ weights adjusted to body weight (mg/kg) for each group of adolescent history. Data are displayed as mean ± SEM.

**Table 3:** RT-PCR data for the paraventricular nucleus and hippocampus.

Adolescent History	Water	10 % Ethanol	10 % Ethanol + 25 μg/mL CORT	10 % Ethanol + 50 μg/mL CORT	10 % Ethanol + 100 μg/mL CORT
Paraventricular Nucleus of the Hypothalamus (PVN)					
GAPDH	107.7 ± 14.9	88.4 ± 4.6	97.0 ± 5.0	93.9 ± 3.6	104.9 ± 8.5
IL-1β	104.7 ± 12.0	126.3 ± 6.0	115.5 ± 12.4	106.6 ± 12.5	109.7 ± 14.7
IL-6	104.8 ± 11.8	105.4 ± 7.1	92.1 ± 7.9	89.8 ± 9.1	98.6 ± 9.1
IκBα	103.5 ± 11.3	133.9 ± 7.4	138.3 ± 9.0	129.1 ± 7.0	125.8 ± 10.9
Iba1	102.6 ± 8.6	120.4 ± 5.7	119.1 ± 5.4	112.6 ± 4.0	102.4 ± 6.4
GFAP	104.0 ± 11.2	131.0 ± 9.0	113.9 ± 7.9	107.3 ± 5.3	108.0 ± 10.4
GR	104.7 ± 10.5	128.1 ± 7.8	120.2 ± 4.6	118.8 ± 6.0	116.2 ± 5.9
MR	104.7 ± 11.1	126.3 ± 5.0	129.9 ± 6.9	124.0 ± 5.8	110.5 ± 7.6
Hippocampus (HPC)					
GAPDH	102.8 ± 7.7	81.8 ± 10.2	108.1 ± 7.0	103.1 ± 13.2	81.6 ± 8.0
IL-1β	105.3 ± 11.2	126.2 ± 20.5	74.2 ± 5.5	96.7 ± 14.0	112.6 ± 10.7
IL-6 *	109.4 ± 15.1 A	175.8 ± 13.2 AB	130.5 ± 18.4 AB	149.3 ± 25.5 AB	188.7 ± 22.3 B
IκBα	103.6 ± 9.2	145.4 ± 20.0	111.4 ± 9.2	113.6 ± 16.0	139.7 ± 8.8
Iba1	103.7 ± 10.3	133.8 ± 16.8	101.4 ± 10.0	97.8 ± 9.1	130.9 ± 10.8
GFAP	104.4 ± 10.3	86.8 ± 4.7	94.7 ± 8.9	90.8 ± 5.5	83.2 ± 5.9
GR	102.7 ± 8.0	123.9 ± 20.5	103.0 ± 8.1	109.8 ± 11.9	133.8 ± 11.7
MR	101.4 ± 5.6	118.1 ± 10.0	104.4 ± 7.7	114.5 ± 9.1	109.5 ± 7.8

Data in this table present the gene expression changes for rats in Exp 1. Data are represented as percent change from the ultimate control (female water group) for each group of adolescent history at the PVN and HPC. An asterisk indicates a main effect of group, *p* < 0.05, and letters indicate groups that significantly differed from one another. Data are displayed as mean ± SEM.

**Table 4:** Adult blood-brain barrier permeability following adolescent consumption model.

Sex	Male		Female	
Adolescent History	Water	10 % Ethanol + 25 μg/mL CORT	Water	10 % Ethanol + 25 μg/mL CORT
Nucleus Accumbens #	34.8 ± 2.7	35.3 ± 2.5	31.0 ± 1.3	29.6 ± 1.9
Cingulate Prefrontal Cortex	32.5 ± 2.9	31.9 ± 3.4	28.8 ± 2.2	27.3 ± 1.2
Amygdala	24.9 ± 1.8	30.5 ± 3.6	25.5 ± 2.9	23.9 ± 1.5
BLA	25.8 ± 1.9	31.4 ± 3.8	26.3 ± 3.0	24.7 ± 1.6
MeA	23.1 ± 1.5	28.5 ± 3.1	23.3 ± 2.6	22.6 ± 0.9
CeA	22.3 ± 1.8	27.6 ± 3.0	23.7 ± 3.1	21.7 ± 1.5
Hippocampus	29.3 ± 2.9	25.2 ± 1.6	26.3 ± 1.8	27.3 ± 1.9
CA1	26.3 ± 2.4	24.5 ± 1.6	24.9 ± 1.6	26.0 ± 1.9
CA2	37.6 ± 4.1	28.0 ± 1.7	30.7 ± 2.7	31.0 ± 1.9
CA3	36.4 ± 4.6	27.2 ± 1.7	29.3 ± 2.8	30.6 ± 2.3
DG	27.4 ± 2.8	23.8 ± 1.4	24.7 ± 1.6	26.0 ± 1.8

Data in this table represent the relative fluorescence units (RFU) of the nucleus accumbens, cingulate prefrontal cortex, amygdala and associated subregions (basolateral, medial, and central amygdala), and hippocampus and associated subregions (CA1, CA2, CA3, and dentate gyrus). Increased RFU indicates increased BBB permeability.

# indicates a main effect of sex, *p* < 0.05.

## Data Availability

Data will be made available on request.
